# Encoding of female mating dynamics by a hypothalamic line attractor

**DOI:** 10.1038/s41586-024-07916-w

**Published:** 2024-08-14

**Authors:** Mengyu Liu, Aditya Nair, Nestor Coria, Scott W. Linderman, David J. Anderson

**Affiliations:** 1 Division of Biology and Biological Engineering, California Institute of Technology, Pasadena, CA USA; 2 Tianqiao and Chrissy Chen Institute for Neuroscience Caltech, Pasadena, CA USA; 3 Howard Hughes Medical Institute, Chevy Chase, MD USA; 4 Department of Statistics, Stanford University, Stanford, CA USA; 5 Wu Tsai Neurosciences Institute, Stanford University, Stanford, CA USA

**Keywords:** Sexual behaviour, Dynamical systems

## Abstract

Females exhibit complex, dynamic behaviours during mating with variable sexual receptivity depending on hormonal status^1–4^. However, how their brains encode the dynamics of mating and receptivity remains largely unknown. The ventromedial hypothalamus, ventrolateral subdivision contains oestrogen receptor type 1-positive neurons that control mating receptivity in female mice^5,6^. Here, unsupervised dynamical system analysis of calcium imaging data from these neurons during mating uncovered a dimension with slow ramping activity, generating a line attractor in neural state space. Neural perturbations in behaving females demonstrated relaxation of population activity back into the attractor. During mating, population activity integrated male cues to ramp up along this attractor, peaking just before ejaculation. Activity in the attractor dimension was positively correlated with the degree of receptivity. Longitudinal imaging revealed that attractor dynamics appear and disappear across the oestrus cycle and are hormone dependent. These observations suggest that a hypothalamic line attractor encodes a persistent, escalating state of female sexual arousal or drive during mating. They also demonstrate that attractors can be reversibly modulated by hormonal status, on a timescale of days.

## Abstract (web summary)

An oestrus cycle-dependent hypothalamic line attractor is discovered to encode the dynamics of female mating behaviour.

## Main

Mating is a complex social interaction whose success is essential to the survival of a species. In rodents, female mating receptivity has been considered as a binary behaviour defined by lordosis^7–10^, a reflexive acceptance posture. In fact, however, female receptivity is highly dynamic, exhibiting variability both within a mating interaction and across different physiological states^2^. Nevertheless, the important contribution of a female to the dynamics of successful mating has been underappreciated and understudied, relative to the male.

Recent progress has identified circuits that control female receptivity^1,3,4,11^. The ventrolateral subdivision of the ventromedial hypothalamic nucleus (VMHvl) contains a subset of *Esr1*^+^ neurons that controls mating behaviours in female mice^5,6,12–15^. Recent findings have revealed hormone-dependent changes in the anatomy and physiology of these neurons. The axonal arborizations of VMHvl progesterone receptor (*Pgr*)-expressing neurons in the anteroventral periventricular nucleus (AVPV) increase in receptive females, in an oestrogen-dependent manner^16^. In addition, a small subset of *Esr1*^+^ neurons, defined by expression of the cholecystokinin A receptor (*Cckar*)^6,17^, has been shown to be necessary and sufficient for female receptivity and to exhibit oestrus cycle-dependent changes in excitability ex vivo and in response dynamics during the investigation phase of mating interactions in vivo^6^. Although these studies have identified important circuit-level changes associated with the state of receptivity, how the dynamics of female behaviour during mating are encoded in the brain is largely unknown.

To address this issue, we have characterized neural population representations in female VMHvl during interactions with males across the oestrus cycle, using longitudinal miniscope imaging of calcium activity^18^. We imaged a subpopulation of *Esr1*^+^ neurons that are *Npy2r*^−^ that we called ‘α-cells’, which causally control sexual receptivity^5^; these cells overlap with the aforementioned *Cckar*^+^ cells^6,7^. Unsupervised modelling of VMHvl α-cell activity using a dynamical systems approach^19^ revealed an approximate line attractor in neural state space, which disappeared during non-receptive phases of the oestrus cycle and was hormone dependent. Analysis of female mating behaviour and line-attractor dynamics suggest that the attractor integrates male contact cues and may represent a persistent, escalating internal state of female sexual arousal or receptivity during mating.

## Dynamics of female behaviours in mating

**Fig. 1:**
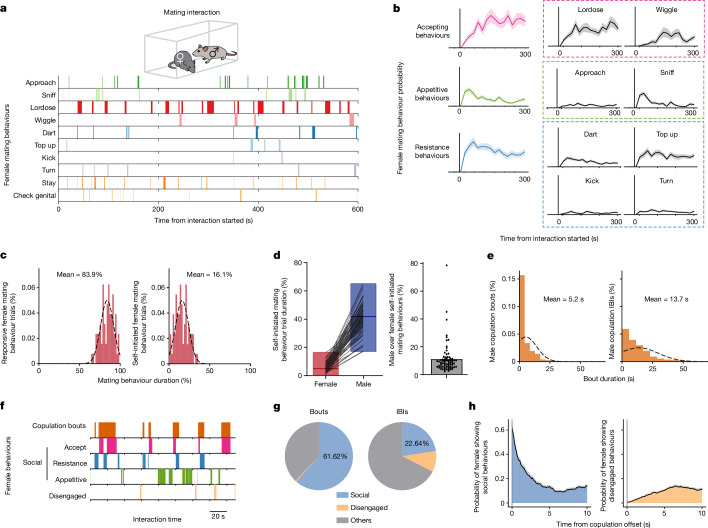
Dynamics of female behaviours during mating interaction. **a**, Raster plot of ten female mating behaviours during one interaction with a male. **b**, The probability of mating behaviours during every 20 s (*n* = 74 trials, *n* = 28 mice). Behaviours were grouped as accept (comprising lordose and wiggle), appetitive (comprising approach and sniff) and resistance (comprising dart, top up, kick and turn); data are presented as mean ± s.e.m. **c**, Distribution of the percentage of time females displayed responsive versus self-initiated mating behaviours over the total mating behaviour time in each trial (*n* = 74 trials). Female self-initiated mating behaviours comprised appetitive behaviours and check genital area. Female-responsive mating behaviours comprised accept and resistance behaviours and staying. **d**, Percentage of time female or male mice displayed self-initiated mating behaviours in each trial (left). The box boundaries range from minimum to maximum, with a line at the median. Male self-initiated mating time over female in each trial (right; *n* = 74 trials). Data are presented as mean ± s.e.m. Male self-initiated mating behaviours included male sniffing, mounting and intromission. **e**, Distribution of the durations of male copulation bouts (left; *n* = 1,685) and IBIs (right; *n* = 1,611). Male copulation included mounting and intromission. **f**, Raster plot of female behaviours during copulation bouts and IBIs. Social behaviours comprised accepting, resistance and appetitive behaviours; and non-social disengaged behaviours comprised rearing, digging and chewing. **g**, Percentage of time females displayed social behaviours in each male copulation bout or IBI. ‘Others’ indicates all behaviours other than the defined social behaviours or non-social disengaged behaviours during interaction. **h**, Female behaviour probability aligned to male copulation offsets.

**Extended Data Fig. 1:**
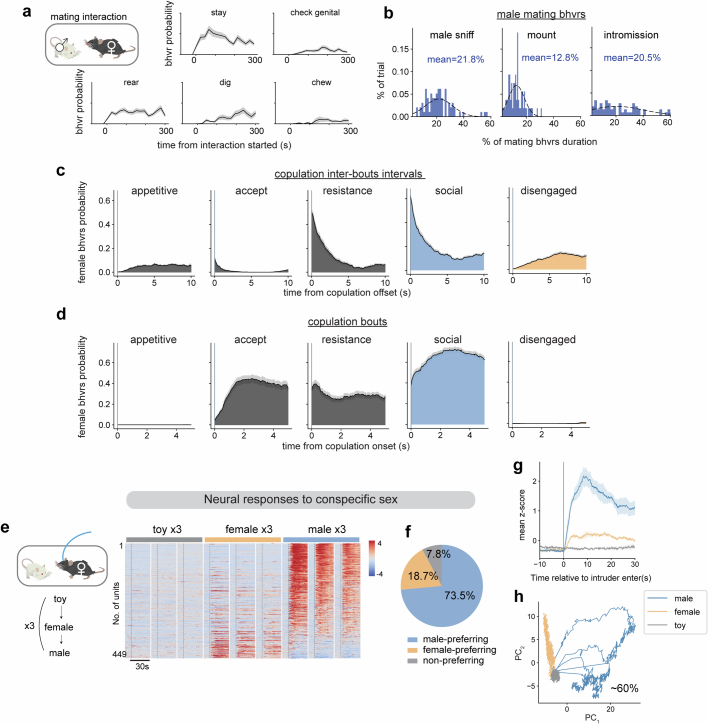
Behavior dynamics and neural responses to conspecific sex. **a**, The probability of female behaviors every 20 s (n = 74 trials, N = 28 mice). **b**, Distribution of the percentage of time males displayed mating behaviors in each trial (n = 74 trials). **c**, The probability of female behaviors aligned to male copulation offsets and **d**, copulation onsets. (**e**-**h**), Neural responses to conspecific sex. **e**, Left, diagram of sex representation test. Each intruder was presented for 1 min. Right, concatenated average responses to toy, female, or male (N = 8 mice). Color scale indicates z-scored activity. Units were sorted by temporal correlation. **f**, Percentages of male- or female-preferring cells (calculated by Choice Probability). **g**, Mean responses of female VMHvl^Esr1^ α cells to male, female and toy (N = 8 mice). Data presented as mean ± SEM. **h**, PCA of neuronal responses to male, female and toy from one example female.

Female mating behaviour has been studied more extensively in rats than in mice^4,20^. To detail mouse female mating behaviour under our standard conditions, we manually annotated video recordings of sexually receptive females interacting with a male (Fig. 1a). We identified ten female motor behaviours and classified them as appetitive (approaching and sniffing the male), accepting (lordosis and wiggling) or resisting (darting, top up, kicking and turning), based on the apparent intent of the behaviour^6^. The behaviours were dynamic, with the probability of accepting behaviours gradually increasing, whereas resistance behaviours initially increased and then slowly decreased (Fig. 1b and Extended Data Fig. 1a). Thus, receptivity is not binary but graded and dynamic.

We categorized mating behaviours as ‘self-initiated’ (appetitive and check genital area) or ‘male-responsive’ (accepting, resistance and staying in response to male attempts). Females spent six times longer displaying male-responsive (83.9%) versus self-initiated behaviours (16.1%; Fig. 1c). Owing to this asymmetry, we also quantified male-initiated behaviours (sniffing, mounting and intromission). Male spent 11.3 times more time displaying self-initiated mating behaviours than females (Fig. 1d), indicating that males largely drive mating interaction. The number and duration of male copulation bouts and interbout intervals (IBIs) varied across interactions (Fig. 1e and Extended Data Fig. 1b).

Next, we compared female behaviours during male copulation bouts versus IBIs (Fig. 1f). Behaviours were classified as ‘social’ (accepting, resisting and appetitive) or ‘disengaged’ (rearing, digging and chewing). During copulation bouts, females primarily exhibited social behaviours (62% of the duration for each bout) and rarely disengaged behaviours (Fig. 1g, left). During IBIs, when females were separated from the males, they continued social behaviours initiated during the preceding bout (Fig. 1g, right; 23% of IBI duration). A behaviour probability plot aligned to copulation offset showed that females continued accepting or resisting behaviours and performed appetitive approaches and sniffing during IBIs (Fig. 1h and Extended Data Fig. 1c).

These results demonstrate that female behaviours during mating are dynamic and primarily driven by male-initiated behaviours. The persistence of female social behaviours observed during pauses in copulation (Fig. 1h) suggests a corresponding persistent internal state of mating receptivity or engagement. The ramping dynamics of ‘accepting’ behaviours (Fig. 1b) further suggests that escalation may be a property of this internal state. Persistence and escalation (or ‘scalability’) are features of internal states underlying other dynamic social behaviours, such as male aggression^21^. We next investigated how these state properties are instantiated by neural activity and dynamics.

## Tuning of female VMHvl neurons in mating

**Fig. 2:**
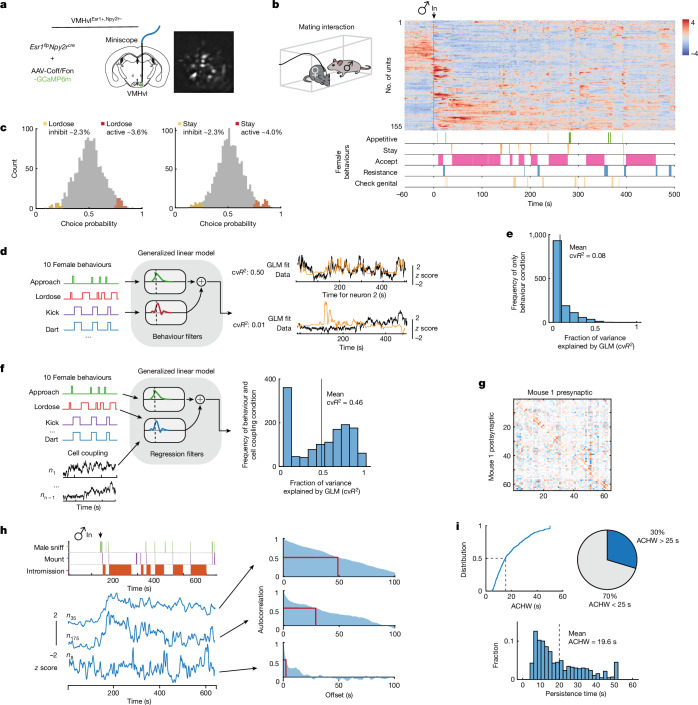
Tuning properties of female VMHvl neurons during mating. **a**, Schematic of the miniscope imaging of female VMHvl^*Esr1+,Npy2r−*^ α-cells (left), and an example imaging plane (right). **b**, Diagram showing the mating interaction test. Single-cell responses during the mating interaction (top right) and their corresponding behaviours (bottom right), from one example female are shown. Units were sorted by temporal correlation. The colour scale indicates *z*-scored activity. **c**, Choice probability histograms and the percentages of tuned cells. Cut-off of choice probability > 0.7 or choice probability < 0.3 and more than 2*σ* (*n* = 15 mice). **d**, Schematic showing the GLM used to predict neural activity from behaviour (left), and an example fit of selected neurons with cv*R*^2^ (right; 0.50 and 0.01). **e**,**f**, Distribution of cv*R*^2^ across all mice for GLMs trained using only behaviour (**e**; *n* = 15 mice) and using behaviour with cell coupling (**f**; *n* = 15 mice). **g**, Predicted cell coupling (relative strength of connectivity) between neurons in one example mouse. **h**, Example VMHvl neurons in female mice showing a range of persistent activity (left; *z*-scored ∆*F*/*F*), and the ACHW as a measure of persistent activity for example units shown (right). The red rectangle highlights the autocorrelation half-width, which is measured by finding the offset value (in seconds) that relates to an autocorrelation of 0.5. **i**, Cumulative distribution of ACHW for all units (top), and distribution of the number of neurons with ACHW > 25 s (bottom; *n* = 4 mice).

To uncover how the dynamics of female mating behaviour are encoded in neural activity, we imaged VMHvl^*Esr1+,Npy2r−*^ (α) cells^5^ in freely moving sexually receptive females interacting with males, using a miniature head-mounted microscope^18^ (Fig. 2a). Initially, we analysed responses observed in the first minute of exposure to either a male or female conspecific and observed distinct subsets tuned to intruder sex, with male-preferring cells approximately four times more abundant, contributing to clear separation of sex in principle component space (Extended Data Fig. 1e,f,h). Correspondingly, the averaged population response to a male was approximately four times higher than that to a female (Extended Data Fig. 1g), consistent with previous bulk calcium imaging studies^5^.

**Extended Data Fig. 2:**
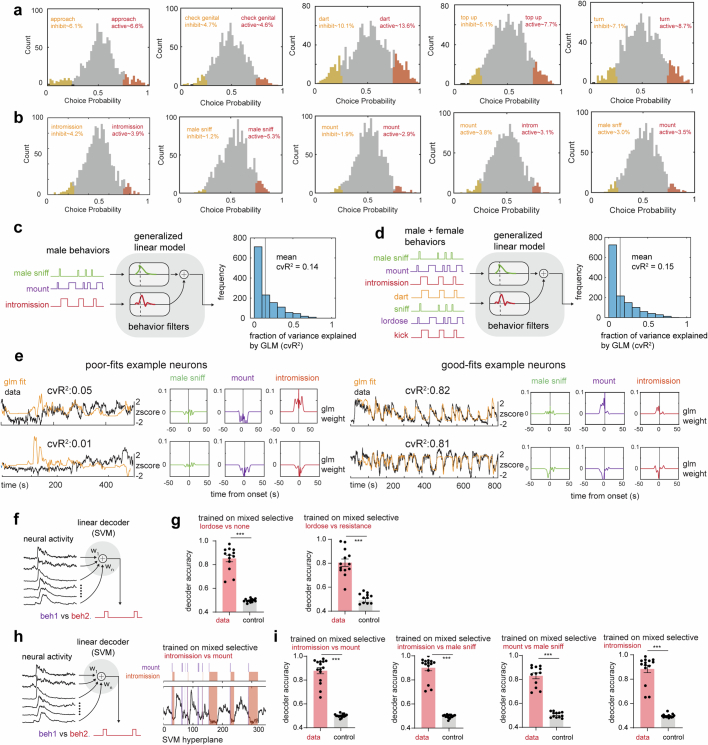
Neural tuning to conspecific sex and behavior. **a**, Choice Probability (CP) histograms and percentages of tuned cells for female behaviors. cutoff: CP > 0.7 or <0.3 and >2σ. N = 15 mice. **b**, Same as **a**, but for male behavior. **c**, Schematic showing generalized linear model (GLM) used to predict neural activity from male behaviors and distribution of cvR^2^ across all mice, or **d**, both male and female behaviors and distribution of cvR^2^ across all mice (N = 15 mice). **e**, Example generalized linear model fits and behavior filters for poorly and well fit neurons. (**f**-**i**), Decoder analysis. **f**, schematic showing linear support vector machine (SVM) decoder trained on frames of male mating behaviors. **g**, performance of the decoder trained to separate female behavior. Left, performance of decoder trained to separate frames of lordosis versus all remaining frames (***p < 0.001, N = 15 mice, mean decoder performance data: 0.85 ± 0.03, shuffle: 0.49 ± 0.003). Right, performance of decoder trained to separate frames of lordosis versus resistance behaviors (***p < 0.001, N = 15 mice, mean decoder performance data: 0.80 ± 0.03, shuffle: 0.48 ± 0.01). **h**, Same as f, but showing the decoder hyperplane for separating male behaviors (mount versus intromission) on right. (***p < 0.001). (N = 15 mice). **i**, performance of decoders trained to separate intromission versus mount (mean decoder performance data: 0.89 ± 0.02, shuffle: 0.49 ± 0.003), intromission versus male sniffing (mean decoder performance data: 0.90 ± 0.02, shuffle: 0.49 ± 0.003), mount versus male sniffing (mean decoder performance data: 0.83 ± 0.02, shuffle: 0.50 ± 0.006) and intromission versus remaining frames male sniffing (***p < 0.001, N = 15 mice, mean decoder performance data: 0.88 ± 0.03, shuffle: 0.48 ± 0.003).

We next analysed imaging data from females acquired during free mating interactions with a male, over 5–10-min observation periods (Fig. 2b; 16–207 units per mouse, mean of 89 ± 12 units per mouse; *n* = 15 mice). Choice probability^22^ indicated that a relatively small percentage of VMHvl α-cells (approximately 2–13%) were ‘tuned’ to specific mating behaviours (Fig. 2c and Extended Data Fig. 2a,b), whereas the majority exhibited ‘mixed selectivity’ (Fig. 2c and Extended Data Fig. 2a,b, grey bars), indicating relatively weak behaviour-specific tuning at the single-neuron level.

To further investigate the relationship between mating behaviours and neural activity, we fit generalized linear models (GLMs) to each neuron using female mating behaviours (Fig. 2d), male mating behaviours (Extended Data Fig. 2c), or both female and male behaviours (Extended Data Fig. 2d). Across all animals, only approximately 8% of the variance in neural activity was explained by female mating behaviours (Fig. 2e; mean cross-validated *R*^2^ (cv*R*^2^) = 0.08; *n* = 15 mice); approximately 14% by male mating behaviours (Extended Data Fig. 2c; mean cv*R*^2^ = 0.14) and approximately 15% of the variance were explained by combined female and male behaviours (Extended Data Fig. 2d; mean cv*R*^2^ = 0.15).

Together, these single-cell analyses indicated that a large fraction of the variance in VMHvl α-cell neural activity during female mating could not be explained by behaviour using a GLM. Nevertheless, a trained support vector machine linear decoder could distinguish mating behaviours with an accuracy higher than chance (Extended Data Fig. 2f–i), suggesting some relationship between neural activity or dynamics and behaviour. To examine whether local interactions between neurons could improve the fit of our GLMs, we included coupling filters^23,24^ in addition to the behavioural variables (Fig. 2f). The introduction of neuronal coupling dramatically increased variance explained by the GLM, suggesting that local circuit interactions contribute more than behaviour to neuronal variance in VMHvl α-cell activity (Fig. 2f,g; mean cv*R*^2^ = 0.46; *n* = 15 mice). Because GLMs fit using low-dimensional coupling matrices (as obtained here) can reflect slowly evolving neural dynamics, we were then motivated to analyse the dynamics of VMHvl α-cell activity.

## Neural dynamics in female VMHvl

**Extended Data Fig. 3:**
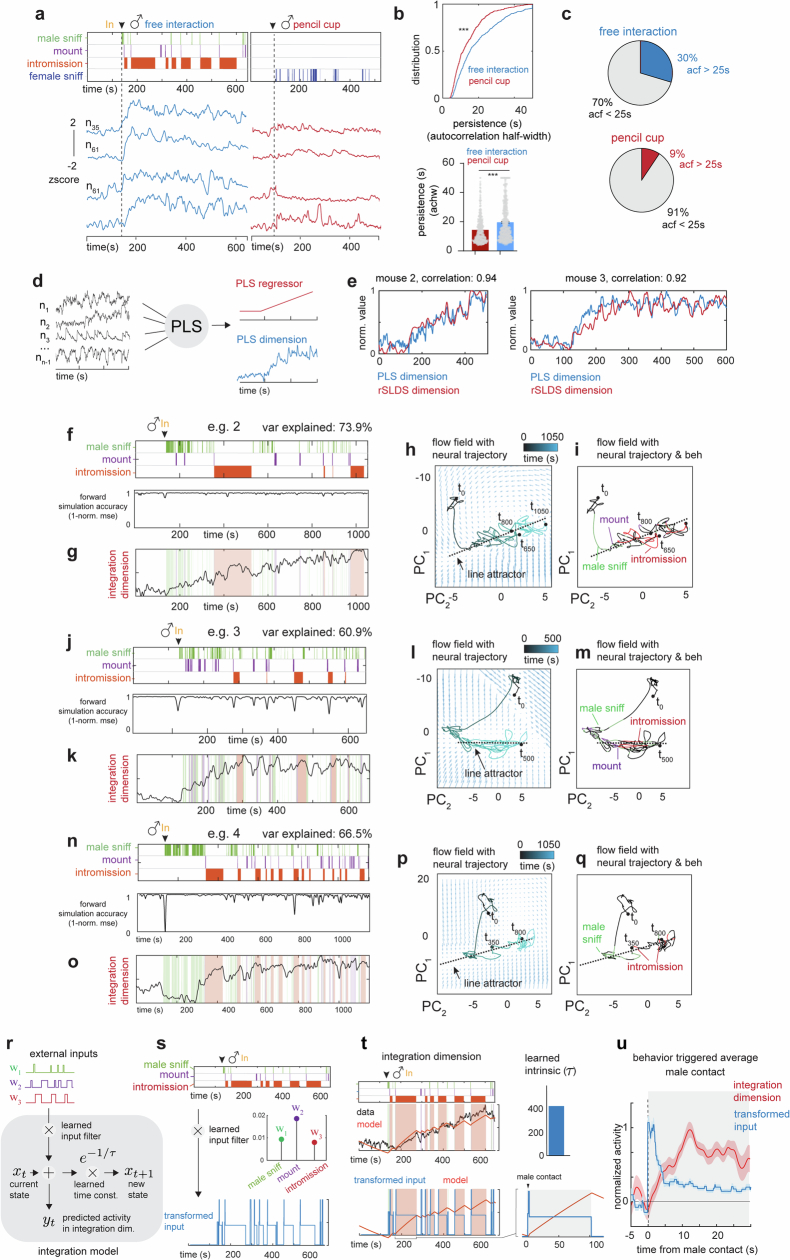
Additional example trials with rSLDS model fit, additional information for Fig. 3. **a**, Dynamics of persistently active neurons identified during receptive interaction with pencil-cup assay. **b**, Cumulative distribution & bar plot of ACHW for same neurons during free interaction vs pencil cup assay ****p < 0.0001, Mann-Whitney U test, p value: 1.25e-11, N = 470 neurons from 5 mice. mean ACHW during pencil cup: 14.3 ± 0.42, free interaction: 19.6 ± 0.58. **c**, Pie chart indicating fraction of neurons with ACHW > 25 s in free interaction and in pencil cup assay. **d**, Schematic illustrating partial least squares regression to extract integration dynamics in VMHvl. **e**, Comparison of rSLDS integration dimension and PLS dimension for two example mice showing a high correlation. (**f**-**q**), Additional example trials with rSLDS model fit. **f**, Recurrent switching linear dynamical systems (rSLDS) model fit forward simulation accuracy aligned to male behaviors in example trial 2. **g**, Dynamics of the integration dimension in trial 2. **h**, Flow field of VMHvl α dynamical system showing neural trajectories in state space, annotated by time from male encounter (t_0_) for trial 2. **i**, Neural state space of VMHvl α dynamical system highlighting behaviors and the region containing the line attractor for trial 2. **j**-**m**, the same as **g**-**i** for example trial 3. **n**-**q**, the same as **j**-**i** for example trial 4. **r**, Integration model used to dissect the contribution of intrinsic decay and external inputs (male behaviors; male-sniff, mount, and intromission). A single state LDS model is used to fit external inputs to predict activity in the integration dimension. **s**, Top: External inputs to integration model, middle: learned input filter showing weights that are multiplied with the external inputs. Bottom: transformed input obtained by multiplying external inputs with input filter. **t**, Top: Data and model prediction from LDS to predict activity in the integration dimension. The learned model has a large intrinsic time constant (right). Bottom: Transformed input (weighted input from three male behaviors) and model prediction overlayed with behaviors. **u**, Behavior triggered average of transformed input and integration dimension aligned to male contact. Male contact is present for the duration of the shaded region. Data presented as mean ± SEM.

We first examined the dynamics of single-neuron activity by measuring the half-width of the autocorrelation (ACHW) function^22^ of each neuron, which is an approximation of the time constant of the neuron^25,26^ (Fig. 2h). This analysis identified individual cells that exhibited apparent persistent activity across the mating interaction. Of cells, 30% displayed ACHWs longer than 25 s (mean ACHW of 20 s; Fig. 2i), a duration longer than the mean copulation IBI (13.7 s; Fig. 1e, right). Of note, the ACHW of the same female cells was significantly lower when the male was confined in a perforated enclosure (pencil cup) than during free mating interaction (mean ACHW for pencil cup of 14.3 ± 0.42 s, mean ACHW for free interaction of 19.64 ± 0.58 s; Extended Data Fig. 3a–c), suggesting that the latter cannot be fully explained by persistent male odours.

**Fig. 3:**
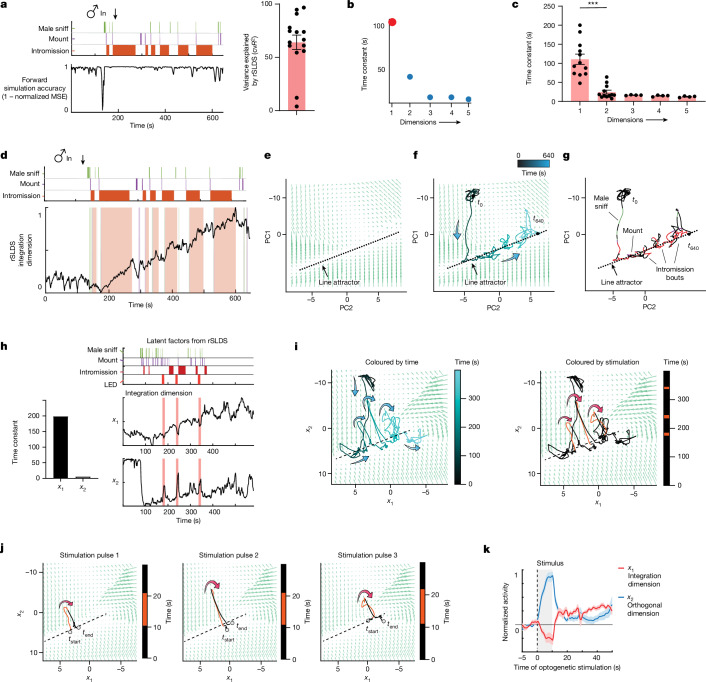
An approximate line attractor in female VMHvl during mating. **a**, rSLDS model performance measured by forward simulation accuracy (calculated as (1 − normalized mean squared error (MSE))^21^ in an example mouse (left), and variance explained by a rSLDS model fit without an input term (Methods) for all mice (right; *n* = 15 mice, mean = 64.08%). The variance explained by the two outliers can be increased by incorporating an input term. **b**, Time constants reveal a single dimension with a large time constant. **c**, Distribution of time constants across animals fit by the rSLDS. Time constants are sorted by magnitude in each animal (***P < 0.001; *n* = 15 mice; mean time constant of dimension 1: 110.7 ± 13.6 and dimension 2: 24.5 ± 5.1; *P* = 6.5 × 10^−5^). **d**, Dynamics of the integration dimension reveals a ramping dimension, aligned to male mating behaviours in an example trial. Variance explained of 73.7%. **e**, Flow field of VMHvl α-cell dynamical system. PC1 principal component 1. **f**, Flow field of VMHvl α-cell dynamical system showing neural trajectories in state space. *t*_0_, time 0 s. **g**, Neural state space of VMHvl α-cell dynamical system and behaviours, highlighting regions where fixed points are present (dashed line). **h**, Time constants of latent factors from the rSLDS model (left), and projection of rSLDS latent factor activity from the rSLDS model trained on neural data from unperturbed periods (right; that is, excluding LED stimulation and 20-s post-stimulation interval). **i**, Flow field and neural trajectories from the rSLDS model coloured by time (left), and neural trajectory coloured by stimulation periods (right). **j**, Flow field and neural trajectories for each of the three stimulation periods for mouse 1. Note that trajectories are pushed away from the attractor during stimulation and then return to the line attractor following stimulation offset, as predicted by the flow field. This approach also tests the validity of the extrapolated regions of the flow field uncovered by the rSLDS. **k**, Stimulus-triggered average of response in integration dimension (*x*_1_) and orthogonal dimension (*x*_2_) upon optogenetic stimulation. *n* = 3 mice. The dotted vertical line indicates the onset of the stimulus, and the shaded area represents the duration of the stimulus. The horizontal line indicates the pre-stimulus baseline of normalized activity.

Given that the single-cell analysis revealed evidence of persistent neural activity, we considered whether a systems-level approach could capture low-dimensional features of population neural dynamics. To this end, we fit an unsupervised dynamical systems model (recurrent switching linear dynamical systems (rSLDS))^19,21^ directly to neural data during individual trials (Fig. 3a,d,e; *n* = 15 mice; mean variance explained (calculated as cv*R*^2^ between observed and predicted neural trajectories) of 64.08 ± 6.8%).

Applying rSLDS analysis to VMHvl α-cell activity revealed an ‘integration dimension’ in state S2 with a relatively long time constant, compared with the other dimensions (110.7 ± 13.6 s; Fig. 3b, red dot, and 3c; *n* = 15 mice). Examining the log_2_ ratio of the two longest time constants to calculate a ‘line-attractor score’^21^ revealed that the fit dynamical system contains a line attractor (Fig. 3e), which is aligned in neural state space to the integration dimension. The integration dimension could also be uncovered using supervised targeted dimensionality reduction (Extended Data Fig. 3d,e), confirming that slow integration dynamics is indeed a property of a subset of VMHvl neurons and not dependent on the method used.

Projecting neural activity into the integration dimension revealed ramping activity that began to increase at the onset of sniffing, mounting or intromission (depending on the trial) and that continued to increase across multiple mating bouts and IBIs (Fig. 3d and Extended Data Fig. 3f–q). The continuous ‘ramping up’ in the integration dimension observed over a long timescale during mating is unexpected; it contrasts with studies of bulk calcium activity in female VMHvl^*Cckar*^ neurons, which have shown a unidirectional decrease from the start of mounting until ejaculation^6^.

To quantitatively reveal the variable integrated by the integration dimension, we modelled the dimension as a neural integrator using a single-state linear dynamical system, allowing the model to flexibly use male behaviours (male-sniff, mount and intromission) to move activity along the integrator (Extended Data Fig. 3r). We found that the model fits with high accuracy (cv*R*^2^ of 0.88 ± 0.02) and possess a large intrinsic time constant (more than 200 s), revealing that it does indeed function as an integrator (Extended Data Fig. 3s). To dissect how the different inputs contribute to the model, we obtained the transformed input from the model (Extended Data Fig. 3s) and found that it peaks following every male contact (Extended Data Fig. 3t, bottom). Thus, male-contact-driven input, in combination with sustained input during male-engagement behaviours such as intromission, is used to integrate activity over time (Extended Data Fig. 3t, bottom, and 3u).

**Extended Data Fig. 4:**
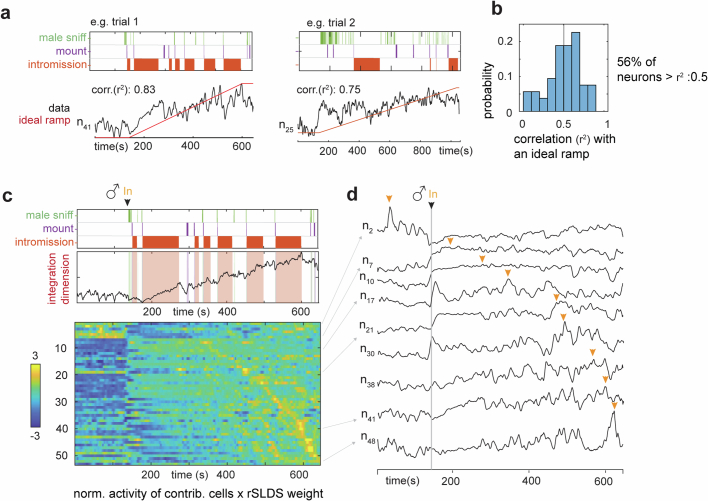
Dynamics of single cell activity. **a**, Correlation of example unit activity with an ideal ramp. **b**, Distribution of correlation of individual neuron activity with ideal ramp. **c**, *Upper*, relationship of male behavior to weighted average of all units contributing to integration dimension as a function of time. Data from the same example trial as shown in Fig. 3d. *Lower*, normalized activity (z-score) of individual units times rSLDS weight for each unit exhibiting a significant weight in the integration dimension, sorted by time to peak. **d**, Traces of example units from **c**, *lower*. Yellow arrow indicates peak of activity for each unit.

Analysis of the traces of individual neurons contributing to the integration dimension indicated that some single cells exhibited ramping-like activity (Extended Data Fig. 4a,b; 56% of neurons *r*^2^ > 0.5), but that different cells peaked at different times during the mating interaction (Extended Data Fig. 4c,d, orange arrowheads). This suggests that the robust ramping activity seen in the integration dimension (Extended Data Fig. 4c, top) is a property of the population and not solely a collective property of all single neurons.

**Extended Data Fig. 5:**
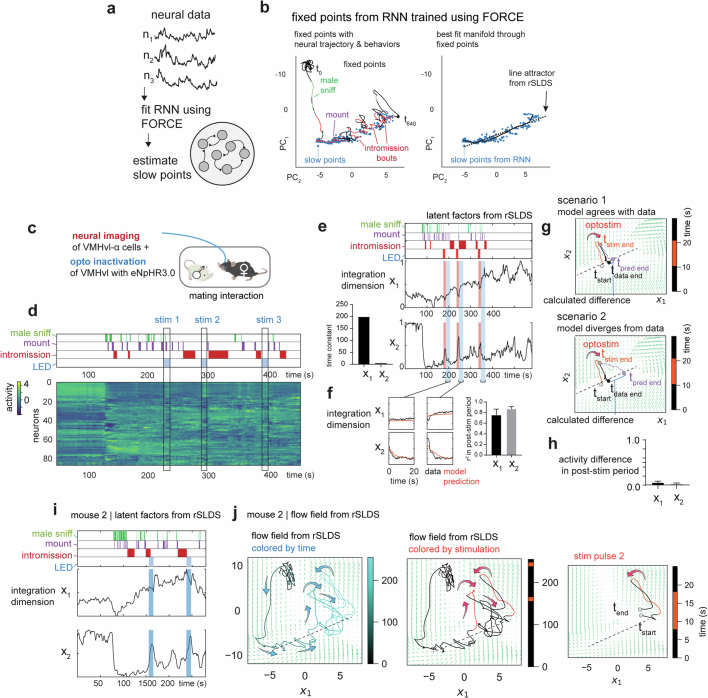
Independent verification and neural perturbations of line attractor dynamics. **a**, Cartoon illustrating approach of fitting RNNs to neural data using FORCE. **b**, Slow points and attractor manifold uncovered by FORCE, overlaid with line attractor uncovered by rSLDS. **c**, Paradigm for simultaneous neural perturbation & imaging during a mating interaction in females. GcaMP was expressed in VMHvl-α cells while halorhodopsin (eNpHR3.0) was expressed in all VMHvl neuron using a pan-neuronal driver. **d**, Neural data obtained from a female showing annotated male behaviors and optogenetic inhibition (LED). **e**, Left: Latent factors from two-dimensional rSLDS model fit to neural data. Reproduced for explanatory purposes from Fig. 3h. Right: Time constants of the two longest-lived dimensions from rSLDS model fit to data from unperturbed periods (excluding stimulation period plus a 20 s post-stimulus period). **f**, Left: Performance of model on held out data from 20 s immediate post-stimulus period (taken from highlighted blue portions of graphs in **e**). **g**, Cartoon depicting quantification of flow field prediction following optogenetic perturbation. The flow field fit from unperturbed periods of time is used to predict the neural trajectory following perturbation (t-pred end, purple line). This trajectory is then compared to data (t-data end, black line). Scenario 1 illustrates when the model agrees with data, resulting in a low difference in activity along the line attractor (top). Scenario 2 illustrates when the model diverges from data resulting in a large deviation in final position along the line attractor (bottom). **h**, Quantification of flow field prediction following perturbation as the difference in activity level at the end of the 20 s post-stimulus period between data and model in both x1 and x2 dimensions across mice (activity difference for x1: 0.05 ± 0.03, for x2: 0.03 ± 0.01, n = 3 mice). **i**, Latent factors from rSLDS of mouse 2 during neural perturbation. **j**, Flow field and neural trajectories for mouse 2. Note that trajectories are pushed away from the attractor during stimulation and then return to line attractor following stimulation offset, as predicted by the flow field.

To visualize the flow field of the rSLDS-fit dynamical system, we projected it into a 2D state space using principal component analysis (Fig. 3e). This projection revealed a stable region (white area) comprising a linear array of ‘slow points’ that approximated a line attractor, which is primarily contributed by the integration dimension of the model (Fig. 3d–g). Mapping annotated behaviours onto the neural trajectory in this state space indicated that the population vector entered the line attractor following initial close contact with the male and progressed along it during successive male intromission bouts (Fig. 3f,g). This progression reflects the ramping seen in the integration dimension discovered by the rSLDS (Fig. 3d and Extended Data Fig. 3f–q). The pattern of fixed points discovered by the rSLDS could also be uncovered by independently fitting recurrent neural networks to neural data using FORCE^27,28^, revealing that the putative line attractor is a feature of neural data and not an artefact of the rSLDS method (Extended Data Fig. 5a,b). Of note, in some animals, the neural vector exhibited brief, loop-like excursions orthogonal to the attractor dimension during IBIs (Fig. 3g and Extended Data Fig. 3i,m,q), suggesting ‘attractiveness’ of the observed fixed points against either natural perturbations orthogonal to the attractor or noise.

## Perturbations of the female line attractor

Definite evidence for the attractive nature of the fixed points discovered by the rSLDS requires performing neural perturbations orthogonal to the line attractor. Such perturbations for line attractors have yet to be performed for freely behaving animals^29^. To achieve this, we performed optogenetic inhibition of the VMHvl network while concurrently imaging VMHvl^*Esr1*^ α-cell neurons (Extended Data Fig. 5c,d). We found that transient optogenetic inhibition creates consistent transient off-manifold responses in neural state space during the period of photostimulation, with the neural trajectory returning to the nearest fixed point along the line attractor post-inhibition (Fig. 3h–k and Extended Data Fig. 5e–j). Using forward simulations of the model fit to data from the unperturbed period (excluding data during and 20 s after stimulation), we found that the dynamical system model is able to predict neural trajectories in the held-out post-stimulus period, revealing the predictive nature of the flow field (Extended Data Fig. 5f–h). Moreover, by providing this inhibition at different positions along the line attractor, we showed that different fixed points along the line attractor revealed by the rSLDS are indeed attractive (Fig. 3i,j and Extended Data Fig. 5i,j).

**Fig. 4:**
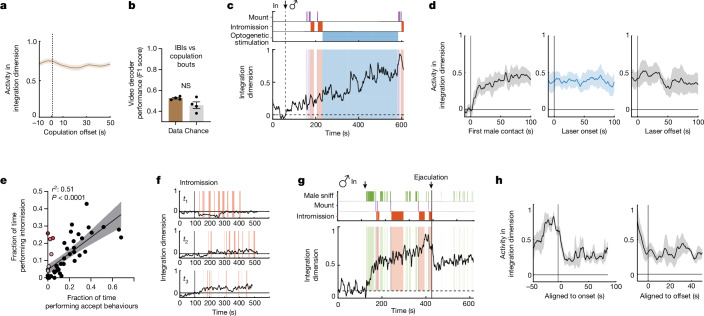
A line attractor encodes a persistent and ramping state during mating. **a**, Behaviour-triggered average of the normalized activity of the integration dimension aligned to the offset of male copulation. Dashed line represents offset of copulation events. Data are presented as mean ± s.e.m. **b**, Videoframe behavioural decoder performance trained on neural data from copulation bouts versus IBIs (*n* = 4 mice, *P* = 0.2, Mann–Whitney *U*-test, mean value of data of 0.52 ± 0.007, shuffle of 0.49 ± 0.03). **c**, Dynamics of the integration dimension in an example female combined with optogenetic inhibition of mating behaviours in the interacting male. **d**, Behaviour-triggered average of the normalized activity of the integration dimension aligned to first male contact (left), optogenetic mating inhibition onset (middle) and inhibition offset (right; *n* = 4 mice). Data are presented as mean ± s.e.m. **e**, Scatter plots of time spent by males performing intromission and time spent by females performing acceptance behaviours to identify trials with high intromission and low receptivity (coloured dots). Data are presented as mean ± s.e.m. *P* = 7.09 × 10^−12^. **f**, Example traces of the integration dimension for trials with intromission but low receptivity (identified from panel **e**). **g**, Dynamics of the integration dimension, aligned to male mating behaviours in an example trial with ejaculation. **h**, Behaviour-triggered average of the normalized activity of the integration dimension aligned to the ejaculation onset and offset (*n* = 4 mice). Data are presented as mean ± s.e.m. Dashed line indicates the onset or offset of ejaculation.

The presence of a line attractor suggested a mechanism to stably maintain population activity in a particular state during interruptions or pauses in male mating behaviour. To test this hypothesis, we first examined activity during copulation IBIs, when the male is physically separated from the female. Of note, we found that the average value of the integration dimension during copulation IBIs was relatively high, similar to and statistically indistinguishable from that measured during the preceding copulation bout (Fig. 4a). Accordingly, it was not possible, using activity in this dimension, to train a decoder to distinguish videoframes containing copulation bouts versus IBIs with accuracy greater than chance (Fig. 4b).

To further probe the stability of the identified line attractor in female VMHvl, we next carried out behavioural perturbation experiments to non-invasively and transiently interrupt male mating (Fig. 4c). After several successful intromission bouts had been performed, we remotely abrogated copulation by optogenetic activation of Sf1^+^ cells from the ventromedial hypothalamus in males, which abruptly promoted a defensive state^30,31^. During the laser-on period, males stopped all mating behaviours, including singing, and displayed no active approach to the female. The induced mating pauses lasted for several minutes (1–5 min), which were much longer than the natural mating pauses (Fig. 1f; average IBI = 13.7 s). Nevertheless, activity in the integration dimension in the female brain remained elevated for minutes while the male was prevented from resuming mating (Fig. 4d), consistent with the persistent activity that we observed during the natural male copulation pauses (Fig. 4a).

Together, these data indicated that VMHvl α-cell activity displays line-attractor dynamics during mating, and that although male contact is integrated along the line attractor (Extended Data Fig. 3r–u), the stability of the system in the integration dimension does not require continuous male contact-dependent sensory input. In further support of this conclusion, in a cohort of naturally cycling females exhibiting variable receptivity (see below), we obtained some trials with high male intromission rates but low female receptivity behaviour (Fig. 4e, coloured dots, and Supplementary Videos 1 and 2). Of note, analysis of those trials revealed relatively little if any ramping in the integration dimension (Fig. 4f). These data suggest that ramping does not simply reflect accumulated mechanosensory inputs derived from male intromission.

Finally, we sought to identify a correlate of the ramping activity revealed by line-attractor dynamics in VMHvl (Fig. 4g). Males displayed sequential mating behaviours with escalating intensity from sniffing to mounting, intromission and finally ejaculation, reflecting an escalating internal state of sexual arousal. We examined activity in the integration dimension during ejaculation and observed that it peaked just before ejaculation, and immediately dropped thereafter (Fig. 4g,h); however, this drop is also characteristic of bulk calcium activity^6^.

## Attractor encodes level of receptivity

**Fig. 5:**
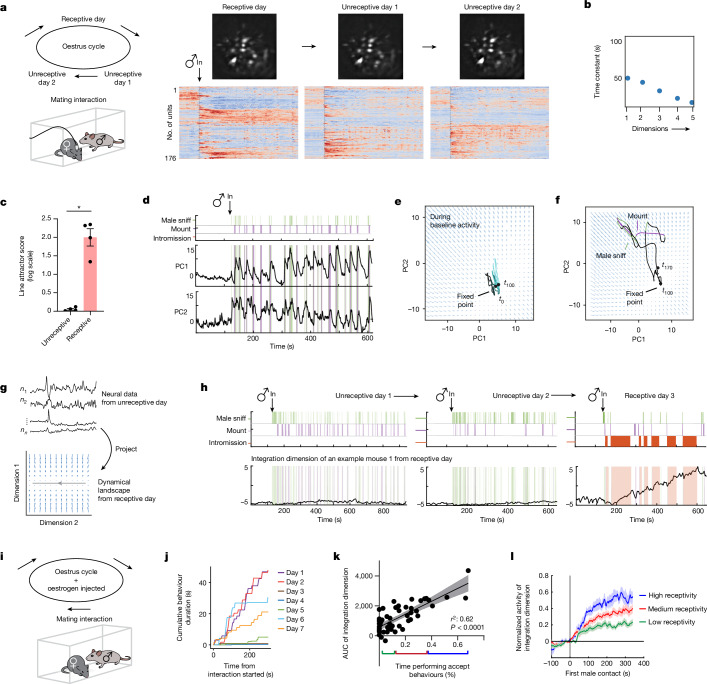
Female line-attractor dynamics encoded sexual receptivity across days. **a**, Illustration of the longitudinal imaging strategy across oestrus states of naturally cycling female mice (left), and example longitudinal imaging planes and traces from one female (right). Units were sorted by temporal correlation. The colour scale indicates *z*-scored activity. **b**, Time constants of the VMHvl α-cell dynamical system on 1 unreceptive day. **c**, Line-attractor scores for dynamical systems fit during receptive and unreceptive days (*n* = 4 mice, mean ± s.e.m. of 0.05 ± 0.02 (unreceptive) and 1.9 ± 0.2 (receptive); *P < 0.05, Mann–Whitney *U*-test, *P* = 0.02). **d**, Low-dimensional principal components of a VMHvl α-cell rSLDS fit model on a unreceptive day. Principal components show fast time-locked dynamics and lack ramping and persistence. **e**, Flow field of the VMHvl α-cell dynamical system on an unreceptive day. **f**, Same as panel **e**, but showing neural trajectories in the state space coloured by time and behaviours. **g**, Schematic showing the projection of neural activity from an unreceptive day into the fit dynamical system from a receptive day. **h**, Dynamics of integration dimension in the VMHvl discovered during a receptive day (same example trial as shown in Fig. 3d) compared with activity of the same dimension on unreceptive days. **i**, Illustration of the longitudinal imaging strategy across oestrus states of naturally cycling females with oestrogen injection. **j**, Accepting behaviours displayed in mating interactions across days from one example female. **k**, Scatter plots of the integration dimension values and the amount of female accepting behaviours (*n* = 50 trials) in each trials. Data are presented as mean ± s.e.m. **l**, The integration dimension activity aligned to the first male contact, in high-receptivity, medium-receptivity or low-receptivity trials, as defined in **k**. ****P < 0.0001, Wilcoxon matched-pairs signed-rank test. Data are presented as mean ± s.e.m.

**Extended Data Fig. 6:**
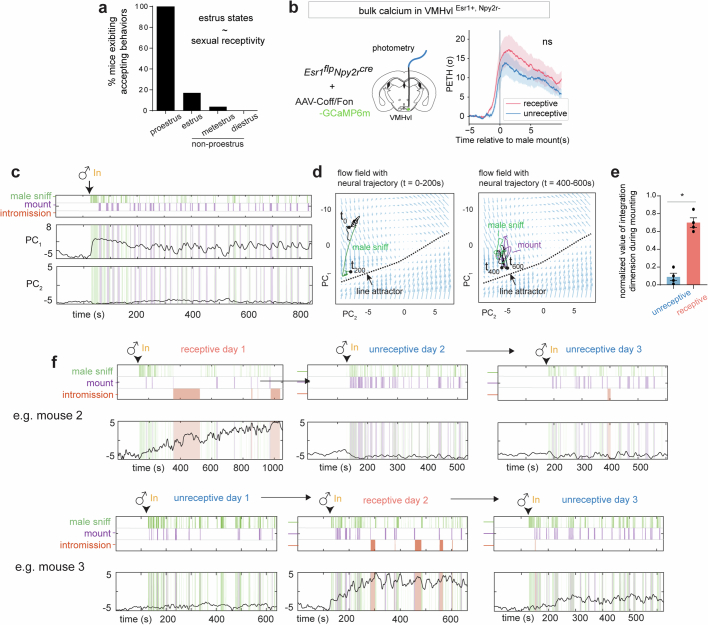
Line attractor dynamics across the estrus cycle. **a**, Correlation between female estrus states and the presence of sexual receptivity, measured by whether female displayed accepting behaviors during interaction with male. **b**, Photometry recording in female VMHvl α cells during receptive and unreceptive mating interactions. Data presented as mean ± SEM. **c**, Low dimensional principal components of VMHvl α dynamical system in receptive day with neural data projected from unreceptive day. **d**, Flow field of VMHvl α dynamical system in receptive day with neural trajectories projected from unreceptive for t = 0 to t = 200 s (left) and t = 200 s to t = 400 s (right). **e**, Quantification of normalized value of integration dimension during male-mounting in unreceptive and receptive days (*p < 0.05, N = 4 mice, mean value during unreceptive day: 0.09 ± 0.04, receptive day: 0.69 ± 0.05. Mann-Whitney U test, p value: 0.02). **f**, Dynamics of integration dimension in two more example mice discovered during receptive day compared to activity of the same dimension on unreceptive days.

We considered whether the line attractor observed during mating reflects or encodes the level of female receptivity by altering receptivity in two different paradigms. First, we performed longitudinal imaging in multiple females (*n* = 4 mice) across their 4–5-day oestrus cycle, during which receptivity changes (Extended Data Fig. 6a). In each animal, we were able to obtain data from 1 sexually receptive day and 2 unreceptive days and to align neurons from those recordings across days (Fig. 5a). Consistent with previous studies^5,6,16^, no change in average VMHvl α-cell population activity (triggered on a male mounting attempt) was apparent on receptive versus unreceptive days (Extended Data Fig. 6b). However, raster plots revealed obvious differences in the pattern of single-unit activity on receptive versus unreceptive days (Fig. 5a, right).

To determine whether there were also differences in VMHvl α-cell dynamics across the oestrus cycle, we fit rSLDS models to data obtained on both receptive and unreceptive days, for each individual. Models fit to data from unreceptive days failed to identify a single dimension with a very long time constant, indicating the absence of a line attractor (Fig. 5b,c). Accordingly, the first two principal components of the rSLDS state space did not exhibit integration-like activity, but rather relatively fast dynamics time-locked to male sniffing and mounting (PC2 in Fig. 5d). In 2D flow-field projections, neural state space contained a single point attractor, reflecting stable baseline activity before interaction with the male, from which the population vector made rapid excursions during sniffing and mounting, returning to the same point attractor after interaction (Fig. 5e,f).

To compare neural dynamics on non-receptive versus receptive days more directly, we projected neural activity from unreceptive days into the rSLDS model fit to data from the receptive day using the same neurons aligned across days (Fig. 5g). The projected neural data failed to show ramping behaviour in the first rSLDS dimension (Fig. 5h and Extended Data Fig. 6c). Accordingly, in 2D projections of the flow field, the neural population activity vector remained at one end of the line attractor (Extended Data Fig. 6d). Although male mounting occurred on unreceptive days (Fig. 5h, purple rasters), activity in the first rSLDS dimension was low during this behaviour (Extended Data Fig. 6e), indicating that it is not sufficient to explain the ramping observed on receptive days.

**Extended Data Fig. 7:**
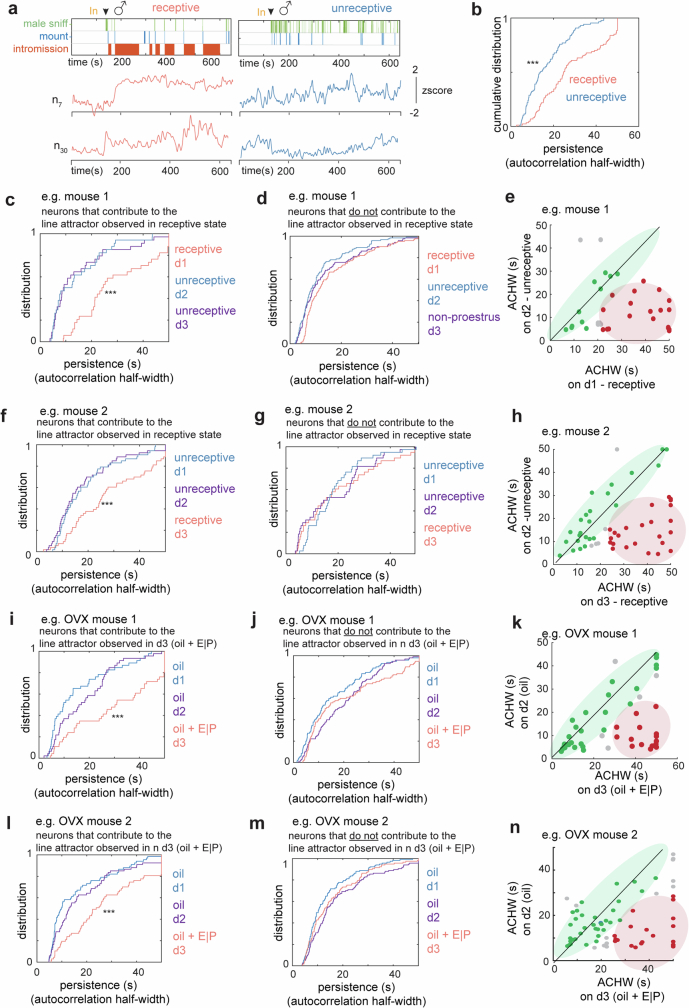
Single cell persistence at receptive and unreceptive days. **a**, Example units active during both receptive (red traces, left) and unreceptive (blue traces, right), showing persistence on receptive day and fast dynamics on the unreceptive days. **b**, Comparison of cumulative distribution of ACHWs to that of same neurons on unreceptive days. Data from example mouse 1. ***p < 0.001, KS-test. **c**, Cumulative distribution of ACHWs for units with significant weights on integration dimension across receptive and unreceptive day, ***p < 0.001, KS-test. Data from example mouse 1. **d**, Cumulative distribution of ACHWs for example mouse 1, for units that do not contribute to the integration dimension on the receptive day, compared on receptive vs unreceptive days. **e**, Scatter plot of ACHWs for units with significant weights on integration dimension for receptive day vs unreceptive day. Data from example mouse 1. (**f**-**h**) Same as **c**-**e** for example mouse 2. **i**, Cumulative distribution of ACHWs for units with significant weights on integration dimension across hormone primed (day 3) and non-primed days (days 2, 1). ***p < 0.001, KS-test. Data from example OVX mouse 1. ***p < 0.001, KS-test. **j**, Cumulative distribution of AHWs for example OVX mouse 1, for units that do not contribute to the integration dimension across hormone primed (day 3) and non-primed days (days 2, 1). **k**, Scatter plot of ACHWs for units with significant weights on integration dimension for hormone-primed day vs non-primed day. Data from example OVX mouse 1 (**l**-**n**) Same as **i**-**k**. for example OVX mouse 2.

**Extended Data Fig. 8:**
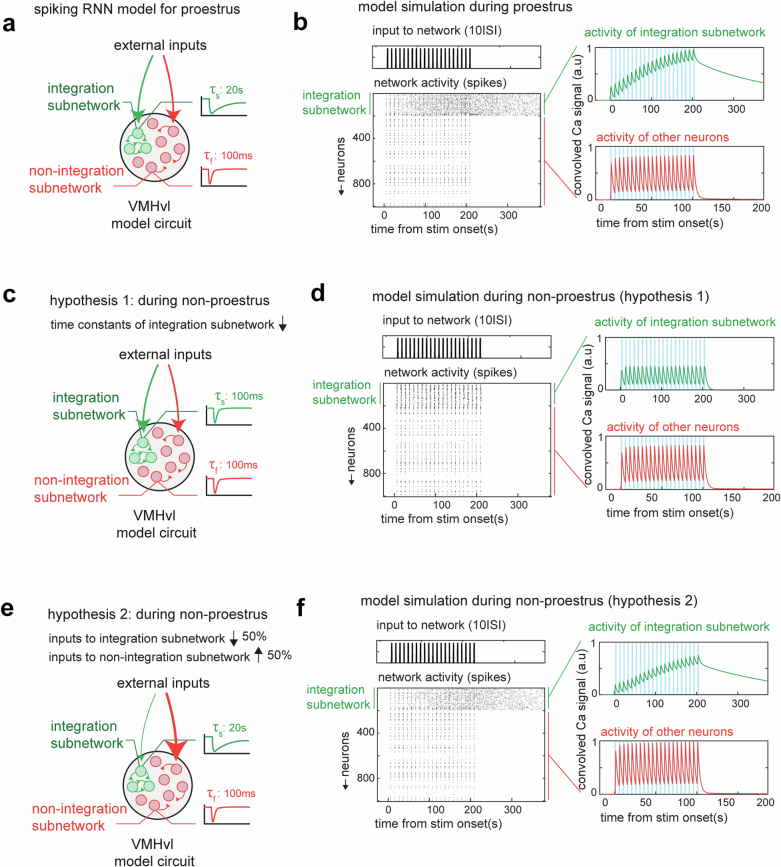
Mechanistic model for loss of line attractor dynamics in unreceptive states. **a**, Schematic illustrating the construction of a spiking recurrent neural network (RNN) with a line attractor. The line attractor is created by allowing a subset of neurons to possess a larger intrinsic time constant (20 s vs 100 ms), and by denser connectivity within the subnetwork (12% versus 1% in remaining network). **b**, Model simulation during the proestrus phase with pulse like input delivered at 10 s ISI. Right, activity of integration subnetwork (green) and other neurons (red). **c**, Schematic for hypothesis 1: we hypothesize that during non-proestrus, there is a reduction in the intrinsic constant of the integration subnetwork (from 20 s to 100 ms). **d**, Same as **b** but for hypothesis 1 during non-proestrus. **e**, Schematic for hypothesis 2: we test whether changes in the firing rate of different neuronal subsets can lead to the loss of attractor dynamics. To investigate this, we provide the integration subnetwork with 50% reduced input strength, while increasing the same for the remaining neurons. **f**, Same as **b** but for hypothesis 2 during non-proestrus.

These results suggested that a change in neural dynamics occurred between receptive and unreceptive days. This inference was supported by the lower ACHW of cells weighted on the first rSLDS dimension on unreceptive versus receptive days (Extended Data Fig. 7a,b; distribution mean for ACHW on unreceptive days 16.1 ± 0.8 s, and on receptive days 25.2 ± 1.5 s; *P* < 0.001). This difference in mean ACHW was observed regardless of the order in which receptive and unreceptive days occurred in different mice (Extended Data Fig. 7c,f). Neurons that did not contribute to the first rSLDS dimension did not exhibit a change in ACHW (Extended Data Fig. 7d,g). Finally, we compared the ACHWs of each individual unit on receptive versus non-receptive days. A scatter plot of these data revealed a subpopulation (39 ± 5%) of line attractor-weighted neurons whose ACHW was higher on receptive than on unreceptive days (Extended Data Fig. 7e,h, red data points). Indeed, incorporating these differences in the ACHW into a mechanistic spiking network model can recapitulate our empirical results, exhibiting a loss of line-attractor dynamics during unreceptive states (Extended Data Fig. 8d).

**Extended Data Fig. 9:**
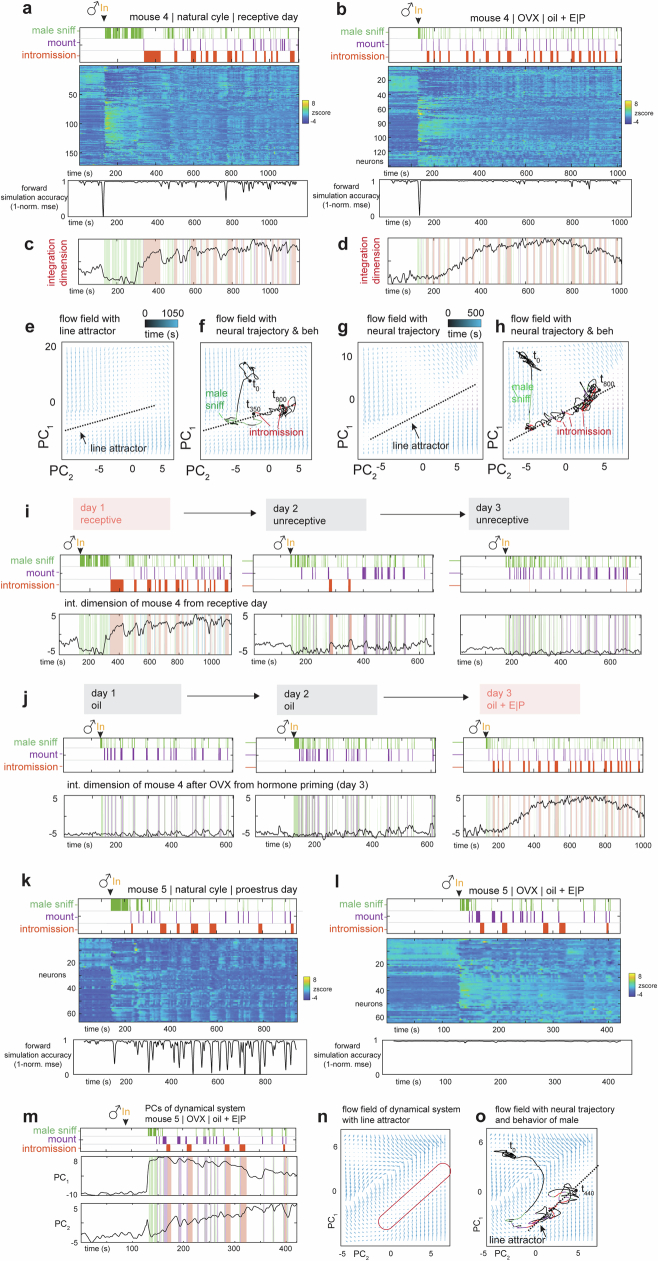
Population dynamics before and after OVX in the same female. (**a**, **b**,) Neural raster and behaviors and rSLDS model performance (measured as forward simulation error, see Methods) for one example mouse in receptive day of natural estrus cycle **a**, and same mouse on hormone primed day after OVX (day3, oil + E | P) **b**. (**c**, **d**), Integration dimension identified by rSLDS on natural cycle receptive day **c**, and during hormone primed day after OVX **d**. (**e**, **f**), Flow field **e**, and neural trajectories of dynamical system **f**, with line attractor highlighted of model fit during the receptive state of the estrus cycle. (**g**, **h**), Same as **e, f**, for model fit during hormone primed day after OVX. **i**, Dynamics of integration dimension discovered during natural cycle receptive day compared to activity of the same dimension on unreceptive days. **j**, Dynamics of integration dimension in the same mouse discovered during hormone primed day (day 3) compared to the activity of the same dimension during non-primed days. (**k**, **l**), Neural raster and behaviors and rSLDS model performance for mouse in proestrus day of natural estrus cycle **k**, and same mouse on hormone primed day after OVX (day3, oil + E | P) **l**. **m**, Principal components of mouse dynamic system fit during hormone primed day. (**n**, **o**), Flow field **n**, and neural trajectories of dynamical system. **o**, with line attractor highlighted of model fit during the hormone primed day after OVX in mouse.

As a second independent test of the hypothesis that the line attractor encodes receptivity, we subjected a cohort of females to ovariectomy (OVX) to render them unreceptive, and performed longitudinal imaging in the OVX animals before versus after hormone priming to restore receptivity (daily injection of oestrogen or progesterone in oil; controls were injected with oil only; see Methods). The results indicated that attractor dynamics disappeared following OVX and were reinstated following hormone priming (Extended Data Fig. 9j). The female mice used in this cohort had also been imaged during their natural cycle and fit with rSLDS models. In some individuals, the model possessed a poor fit on receptive days (Extended Data Fig. 9k, ‘forward simulation accuracy’). In one such animal, the fit of the rSLDS model was markedly improved following OVX and hormone priming, compared with the fit obtained on her naturally receptive day (Extended Data Fig. 9k versus 9l–n). Together, these data confirm a strong prediction of the hypothesis that the line attractor observed during mating encodes some aspect of mating receptivity.

**Extended Data Fig. 10:**
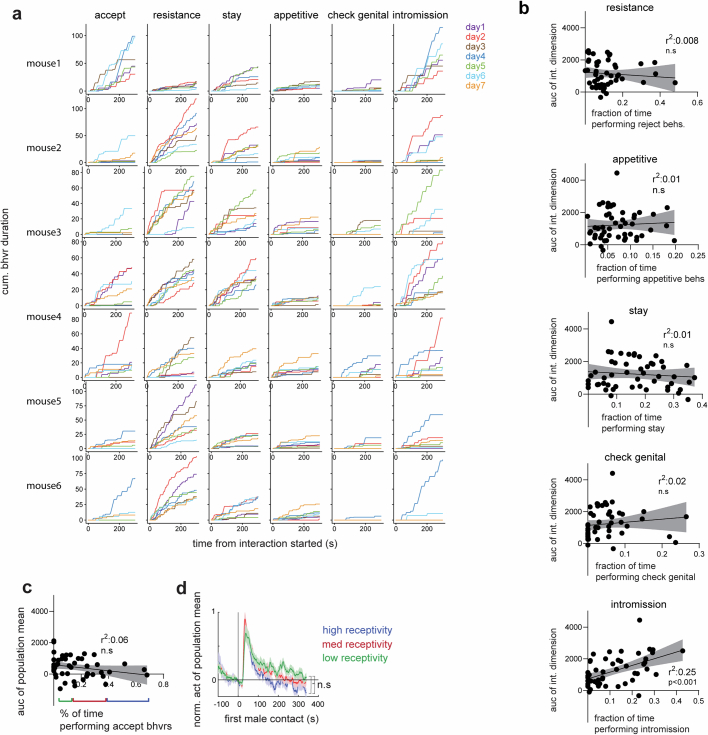
Longitudinal mating assay and correlation with attractor dynamics. **a**, Behaviors displayed in mating interactions across days from all the recorded females. **b**, The scatter plots of the integration dimension values and the amount of female resistance behaviors (linear regression, R^2^ = 0.008), appetitive behaviors (R^2^ = 0.01), staying (R^2^ = 0.01), checking genital (R^2^ = 0.02) and male intromission (R^2^ = 0.25). Data presented as mean ± SEM. **c**, correlation of area under the curve (auc) of the population mean of all neurons with the percentage of time spent performing accepting behaviors. Data presented as mean ± SEM. **d**: activity of population mean from trials with varying degrees of receptivity defined in **a**). Data presented as mean ± SEM, Mann-Whitney U test.

The foregoing data left open the important question of whether the continuous low-dimensional variable instantiated by the line attractor reflects or encodes continuous variation in the degree of female receptivity. In males, differences in the time constant of the integration dimension are strongly correlated with differences in aggressiveness, across individual animals^19^. We therefore sought to examine line attractor parameters within a cohort of females exhibiting individual differences in receptivity across trials and days. To generate this cohort, we injected naturally cycling female mice with oestrogen (but not progesterone) daily beginning 2 days before imaging and continued the injections during 3–7 days of repeated imaging of the same animals during daily mating tests (*n* = 6 mice). These injections increased the number of days on which females exhibited receptivity, while still allowing variation in the level of receptivity (as measured by the amount of accepting behaviours displayed in a given trial) in response to changing levels of endogenous sex hormones across the oestrus cycle (Fig. 5i,j and Extended Data Fig. 10a). This design afforded the opportunity to correlate quantitative variation in receptivity with variation in line-attractor parameters.

We fit rSLDS models to imaging data from each animal and mating trial and plotted the average activity of the integration dimension over time (refer to Fig. 5h). The area under the curve was strongly and positively correlated with the percentage of time that females performed acceptance behaviours during each mating interaction (Fig. 5k,l; *r*^2^ = 0.62; *P* < 0.0001; *n* = 50 trials). By contrast, other behaviours such as resistance and appetitive behaviours were not correlated with this measure (Extended Data Fig. 10b). Finally, the population mean of neural activity did not show any correlation with the percentage of time that females performed acceptance behaviours, highlighting the value of rSLDS to identify physiologically distinct subpopulations whose activity is quantitatively correlated with receptivity during male mating (Extended Data Fig. 10c,d). Thus, these data indicate that variation in movement along the line attractor reflects variation in levels of sexual receptivity, across individuals and trials.

## Discussion

Using unsupervised analysis of neural data, we discovered an approximate line attractor in a genetically defined subset of VMHvl^*Esr1*^ neurons that causally controls female mating receptivity. Activity in the attractor scales with individual differences in receptivity and is oestrus cycle dependent. To our knowledge, no previous example of a line attractor that appears and disappears with periodic changes in behavioural/hormonal state on a timescale of days exists.

Line-attractor dynamics can afford internal states two important features: stability (persistence) and ramping (scalability). The stability of the line attractor across intromission bouts may function to maintain the female in a persistently aroused state during intermittent male copulatory behaviour, facilitating its re-initiation following pauses. This interpretation is supported by our observation that female social behaviour persists following natural interruptions in copulation (Fig. 1f–h). The ramping activity seen during copulation may represent a continuous, scalable variable in the female brain. A reasonable interpretation is that this variable encodes the level of escalating female sexual arousal. We emphasize that this ramp-up was not visible in the bulk α-cell calcium signal, but only in the integration dimension. This may explain why it was not reported in a study of mating-related VMHvl^*Cckar*^ neuronal activity using fibre photometry^6^.

The idea that the line attractor encodes mating receptivity is supported by its presence or absence during receptive versus unreceptive oestrus cycle days or in ovariectomized females with versus without hormone priming, respectively (Fig. 5c). However, it is not just a binary correlate of receptivity: the degree of movement along the attractor was highly correlated (*r*^2^ = 0.62) with the level of receptivity as measured by the frequency of accepting behaviours (Fig. 5k). By contrast, the integration dimension was not well correlated with other female behaviours (Extended Data Fig. 10b).

Our previous work has shown that transcriptionally distinct subsets of VMHvl^*Esr1*^ neurons, called α-cells and β-cells, control female sexual receptivity and maternal aggression, respectively^5^. Here we show that the α-cell population exhibits further heterogeneity at the physiological level, including subsets that contribute to the line attractor or to orthogonal dimensions. Whether these subpopulations are transcriptomically distinct is not yet clear^6,17^. Yin et al.^6^ previously reported that VMHvl^*Cckar*^ neurons (a subset of α-cells) displayed receptivity-associated changes in their spontaneous activity, and responsivity to males during investigation. These cells may contribute to the integration dimension neurons identified here.

Together, our data suggest that neural population dynamics represent the dynamics of female mating receptivity and can be reversibly sculpted by physiological state. They also generalize the concept that line attractors with slow dynamics encode internal states underlying innate social behaviours^21^. Because the molecular, cellular and connectional features of VMHvl are well described^5,14,15,32–34^, this system may be advantageous for understanding how hormones, genes, cell types and local circuitry contribute to emergent neural population dynamics.

## Methods

### Mice

All experimental procedures involving the use of live mice or their tissues were carried out in accordance with NIH guidelines and approved by the Institute Animal Care and Use Committee (IACUC) and the Institute Biosafety Committee (IBC) at the California Institute of Technology (Caltech). All mice in this study, including wild-type and transgenic mice, were bred at Caltech or purchased from Charles River Laboratory. Group-housed C57BL/6N female or singly housed male mice (2–5 months) were used as experimental mice. *Npy2r*^*cre*^ mice (Jackson Laboratory stock no. 029285; *n* = 1), *Esr1*^*cre*^ mice, *Esr1*^*flp*^ mice (*n* > 10) and *Sf1*^*cre*^ mice (Jackson Laboratory stock no. 012462) were backcrossed into the C57BL/6N background and bred at Caltech. Heterozygous *Npy2r*^*cre*^, *Esr1*^*cre*^ or double heterozygote *Esr1*^*flp/+*^*Npy2r*^*cre/+*^ mice were used for cell-specific targeting experiments and were genotyped by PCR analysis using genomic DNA from tail tissue. All mice were housed in ventilated microisolator cages in a temperature-controlled environment (median temperature of 23 °C, humidity of 60%), under a reversed 11-h dark–13-h light cycle, with ad libitum access to food and water. Mouse cages were changed weekly.

### Surgeries

Surgeries were performed on female *Esr1*^*flp*^^/^^+^*Npy2r*^*cre*^^/^^+^ mice 2 months of age. Virus injection and implantation were performed as previously described^35,36^. In brief, mice were anaesthetized with isoflurane (5% for induction and 1.5% for maintenance) and placed on a stereotaxic frame (David Kopf Instruments). Virus was injected into the target area using a pulled-glass capillary (World Precision Instruments) and a pressure injector (Micro4 controller, World Precision Instruments), at a flow rate of 20 nl min^–1^. The glass capillary was left in place for 10 min following injection before withdrawal. Lenses were slowly lowered into the brain and fixed to the skull with dental cement (Metabond, Parkell). Females were co-housed with a vasectomized male mouse after virus injection and lens implantation. Four weeks after lens implantation, mice were head-fixed on a running wheel and a miniaturized micro-endoscope (nVista, nVoke, Inscopix) was lowered over the implanted lens until GcaMP-expressing fluorescent neurons were in focus. Once GcaMP-expressing neurons were detected, a permanent baseplate was attached to the skull with dental cement. The co-housed vasectomized males were removed.

### Virus injection and GRIN lens implantation

The following adeno-associated viruses (AAVs) were used in this study, with injection titres as indicated. Viruses with a high original titre were diluted with clean PBS on the day of use. AAV-DJ-EF1a-Coff/Fon-GcaMP6m (4.5 × 10^12^, Addgene plasmid) was packaged at the HHMI Janelia Research Campus virus facility. ‘Coff/Fon’ indicates Cre-OFF/FLP-ON virus. Stereotaxic injection coordinates were based on the Paxinos and Franklin atlas. Virus injection: VMHvl, anteroposterior −1.6, mediolateral ±0.78, dorsoventral −5.7; GRIN lens implantation: VMHvl: anteroposterior −1.6, mediolateral −0.76, dorsoventral −5.55 (∅0.6 × 7.3 mm GRIN lens).

### Vaginal cytology

To determine the oestrus phases of tested females, vaginal smear cytology was applied on the same day as the behavioural test. A vaginal smear was collected immediately after the behavioural test and stained with 0.1% crystal violet solution for 1 min. Cell types in the stained vaginal smear were checked microscopically. In this study, the pro-oestrus phase was characterized by many nucleated epithelial, some cornified epithelial and no leukocytes.

### Hormone priming

Female mice were ovariectomized and oestrus was induced by hormone priming. Oestradiol benzoate (E2) and progesterone powder were dissolved in sesame oil. For primed females, 50 µl 200 µg ml^−1^ oestradiol benzoate was delivered subcutaneously on days -2 and -1 at 15:00. Then, 10 mg ml^−1^ progesterone was delivered subcutaneously on the day of test at 10:00. The behavioural test was performed 4–6 h after progesterone injection. For unprimed female mice, sesame oil was injected at the same timepoints as hormone injections. Vaginal smear cytology was applied on the same day as the behavioural test to make sure that the females were completely primed or unprimed. For oestrogen-injected females used in Figs. 4 and 5, 50 µl 200 µg ml^−1^ oestradiol benzoate was delivered subcutaneously every day at 10:00. The behavioural tests were conducted after the first 2 days of injection.

### Sex representation assay

All behavioural tests were performed under red light. Group-housed C57BL/6N male and female mice (2–4 months) were used for the test. The tested female was acclimated in her home cage under the recording setup^37^ for 10 min. A toy, a female or a male was introduced to the tested female with a 90-s interval. Each interaction lasted for 1 min before transitioning into the consummatory phase. The sequential representations were repeated three times.

### Mating assay

Singly housed sexually experienced C57BL/6N male mice were used for the mating assay. Male mice used for the test were initially co-housed with a female mouse for at least 1 week and singly housed at least 1 week before the test. On the day of the test, a male mouse was acclimated in his home cage under the recording setup. A random female mouse was placed into male cage until three male mounting bouts were observed. The tested female mice were acclimated in a new cage for 10 min before being introduced into the male cage. The male contact mating interaction lasted for 5–15 min. At the end of the free interaction, a pencil cup was introduced to restrain the male. Then, imaging and behavioural recording during the non-contact period continued for 3–5 min.

### Wireless optogenetic male mating inhibition assay

Singly housed sexually experienced *Sf1*^*cre+/−*^ males were used in this test. All hardware and wireless devices for optogenetic stimulation were sourced from NeuroLux. Specifically, AAV2-EF1a-DIO-hChR2(H134R)-EYFP-WPRE-pA (4.2 × 10^12^, UNC vector core) was unilaterally injected into ventromedial hypothalamus of the male mice at coordinates: anteroposterior −1.5, mediolateral +0.4, dorsoventral −5.6. Simultaneously, wireless optogenetic devices were implanted (NeuroLux). A recovery period of 3 weeks followed the surgical procedures to allow for optimal viral vector expression and to ensure the wellbeing of the mice. Subsequently, a mating assay was performed, and when multiple successful copulations were observed, male mice were exposed to a wirelessly powered blue-light photostimulation (473 nm for 1–5 min at 20 Hz and 10 W). During the stimulation, male mice promptly discontinued all mating-related behaviours, including vocalization, sniffing, mounting or intromission, and instead exhibiting exploratory behaviours within the home cage and distancing themselves from the female mouse. Following the cessation of photostimulation, male mice typically resumed mating-related behaviours, either immediately or with a delay.

### Behavioural annotations

Behavioural videos were manually annotated using a custom MATLAB-based behavioural annotation interface. A ‘baseline’ period of 2 min when the animal was alone in its cage was recorded at the start of every recording session.

During female–male interaction, we manually annotated the following male mating behaviours: male sniff, mount, intromission and ejaculation.

For the same video, we annotated the following female mating behaviours: approach, sniff, lordose, wiggle, stay, dart, top up, kick, turn and check genital. For ‘approach’, the female faced male and walked to it without pausing. For ‘sniff’, the female actively sniffed the male. For ‘lordose’, the female abdomen was on the ground and motionless or showing an arched back posture responding to male mounting or intromission. For ‘wiggle’, the female continuously moved her head or body responding to male mounting or intromission. For ‘stay’, the female quietly stayed in place, but the abdomen was not clearly on the ground, responding to male mounting or intromission. For ‘dart’, the female quickly ran away from male, responding to male mating behaviours. For ‘top up’, the female stood up to conceal the anogenital area, responding to male mating behaviours. For ‘kick’, the female kicked the male, responding to male mating behaviours. For ‘turn’, the female turned away from the male, responding to mating mounting or intromission. For ‘check genital’, the female examined her genital area, usually after male mounting or intromission.

Lordose, wiggle, stay, dart, top up, kick and turn were grouped as responsive mating behaviours. Approach, sniff and check genital were grouped as female self-initiated mating behaviours.

Female approach and sniff were grouped as appetitive mating behaviours. Lordose and wiggle were grouped as accepting mating behaviours. Dart, top up, kick and turn were grouped as resistance mating behaviours.

All appetitive, accepting and resistance behaviours were grouped as social behaviours.

For the same video, we also annotated the following female non-social disengaged behaviours: rear, dig and chew. For rear, the female extended her body upright and attempted to explore outside the testing chamber. For dig, the female dug beddings. For chew, the female stood up and chewed with her mouth.

### Fibre photometry

The fibre photometry setup was as previously described in earlier research with minor modifications. We used 470-nm LEDs (M470F3, Thorlabs, filtered with 470–10-nm bandpass filters FB470-10, Thorlabs) for fluorophore excitation, and 405-nm LEDs for isosbestic excitation (M405FP1, Thorlabs, filtered with 410–10-nm bandpass filters FB410-10, Thorlabs). LEDs were modulated at 208 Hz (470 nm) and 333 Hz (405 nm) and controlled by a real-time processor (RZ5P, Tucker David Technologies) via an LED driver (DC4104, Thorlabs). The emission signal from the 470-nm excitation was normalized to the emission signal from the isosbestic excitation (405 nm), to control for motion artefacts, photobleaching and levels of GcaMP6m expression. LEDs were coupled to a 425-nm longpass dichroic mirror (DMLP425R, Thorlabs) via fibre optic patch cables (diameter of 400 mm, NA of 0.48; Doric lenses). Emitted light was collected via the patch cable, coupled to a 490-nm longpass dichroic mirror (DMLP490R, Thorlabs), filtered (FF01-542/27-25, Sem-rock), collimated through a focusing lens (F671SMA-405, Thorlabs) and detected by the photodetectors (Model 2151, Newport). Recordings were acquired using Synapse software (Tucker Davis Technologies). On the test day, after at least 5 min of acclimation under the recording setup, the female was first recorded for 5 min to establish a baseline. Then, behavioural assays were proceeded and fluorescence was recorded for the indicated period of time, as described in the main text. All data analyses were performed in Python. Behavioural video files and fibre photometry data were time-locked. Fn was calculated using normalized (405 nm) fluorescence signals from 470-nm excitation. Fn(*t*) = 100 × [F470(*t*) – F405fit(*t*)]/F405fit(*t*). For the peri-event time histogram, the baseline value *F*_0_ and standard deviation s.d._0_ were calculated using a −5-s to −3-s window. Overlapping behavioural bouts within this time window were excluded from the analysis. Then, the peri-event time histogram was calculated by [(Fn(*t*) – *F*_0_]/s.d._0_.

### Microendoscopic imaging data acquisition

Imaging data were acquired by nVista 3.0 (Inscopix) at 30 Hz with two times spatial downsampling; LED power (0.2–0.5) and gain (6–8×) were adjusted depending on the brightness of GcaMP expression as determined by the image histogram according to the user manual. A transistor–transistor logic pulse from the Sync port of the data acquisition box (DAQ, Inscopix) was used for synchronous triggering of StreamPix7 (Norpix) for video recording.

For perturbation-imaging experiments, AAV5-hSyn-eNpHR3-mCherry (Addgene) was expressed together with GcaMP in the VMHvl. Imaging data were acquired by nVoke 2.0 (Inscopix). One to three bouts of inhibitory LED stimulation (5 mW continuous for 10 s) were performed during receptive mating trials.

### Microendoscopic data extraction and preprocessing

Miniscope data were acquired at 30 Hz using the Inscopix Data Acquisition Software as two times downsampled .isxd files. Preprocessing and motion correction were performed using Inscopix Data Processing Software. In brief, raw imaging data from the same animal from multiple recording dates were concatenated, two times spatially downsampled, motion corrected and temporally downsampled to 10 Hz further and exported as a .tiff image stack. A spatial bandpass filter was then applied to remove out-of-focus background. After preprocessing, calcium traces were extracted and deconvolved using the CNMF-E large data pipeline with the following parameters: patch_dims = [32, 32], gSig = 3, gSiz = 13, ring_radius = 19, min_corr = 0.75 and min_pnr = 8. The spatial and temporal components of every extracted unit were carefully inspected manually (signal-to-noise ratio, peak-to-noise ratio, size, motion artefacts, decay kinetics and so on) and outliers (obvious deviations from the normal distribution) were discarded. The extracted traces were then *z*-scored before analysis.

### Longitudinal imaging data extraction and preprocessing

The females performed the mating assay and were imaged for consecutive 3–7 days. ‘Receptive day’ was defined as the female displaying accepting behaviours on the testing day, whereas ‘unreceptive day’ was defined as the female not displaying accepting behaviours on the testing day. Miniscope data from one receptive day and two unreceptive days were selected and concatenated to one .isxd file. Data were preprocessed and the traces were extracted as described in the previous section. The 3-day concatenated traces were *z*-scored and then split to multiple traces for individual days.

### Choice probability

Choice probability is a metric that estimates how well either of two different behaviours can be predicted or distinguished, based on the activity of any given neuron during these two behaviours. Choice probability of single neurons was computed using previously described methods^36^. To compute the choice probability of a single neuron for any behaviour pair, 1-s binned neuronal responses occurring during each of the two behaviours were used to generate a receiver operating characteristic curve. Choice probability is defined as the area under the curve bounded between 0 and 1. A choice probability of 0.5 indicates that the activity of the neuron cannot distinguish between the two alternative behaviours. We defined a neuron as being capable of distinguishing between two behaviours if the choice probability of that neuron was more than 0.7 or less than 0.3 and was more than 2 s.d. or 2 s.d. or less of the choice probability computed using shuffled data (repeated 1,000 times).

### GLM

To predict neural activity from behaviour, we trained GLMs to predict the activity of each neurons *k*, as a weighted linear combination of three male behaviours: male sniffing, mounting and intromission:

$${y}_{k}(t)=\mathop{x}\limits^{\rightharpoonup }(t)\mathop{\beta }\limits^{\rightharpoonup }+\varphi $$

Here ${y}_{k}(t)$ is the calcium activity of neuron *k* at time *t*, $\mathop{x}\limits^{\rightharpoonup }(t)$ is a feature vector of three binary male behaviours at time lags ranging from *t*-D to *t* where D = 10 s. $\mathop{\beta }\limits^{\rightharpoonup }$ is a behaviour filter, which describes how a neuron integrates stimulus over a 10-s period (example filters are shown in Extended Data Fig. 2d,e). *φ* is an error term. The model was fit using tenfold cross-validation with ridge regularization, and the model performance is reported as cv*R*^2^. To account for cell–cell interactions within the network, we also used the activity of simultaneously imaged neurons as regressors in addition to behaviour as previously performed^23,24^.

### Dynamical system modelling

rSLDS models^19,38^ are fit to neural data as previously described^21^. In brief, the rSLDS is a generative state-space model that decomposes non-linear time series data into a set of linear dynamical systems, also called states. The model describes three sets of variables: a set of discrete states (*z*), a set of latent factors (*x*) that captures the low-dimensional nature of neural activity and the activity of recorded neurons (*y*). Although the model can also allow for the incorporation of external inputs based on behavioural features, such external inputs were not included in our first analysis.

The model is formulated as follows: at each timepoint, there is a discrete state ${z}_{t}\in \{1,\ldots ,{K}\}$ that depends recurrently on the continuous latent factors (*x*):

1 $$p\left({z}_{t+1}| {z}_{t}=k,{x}_{t}\right)={\rm{softmax}}\left\{{R}_{k}{x}_{t}+{r}_{k}\right\}$$

where ${R}_{k}\in {{\mathbb{R}}}^{K\times K}$ and ${r}_{k}\in {{\mathbb{R}}}^{K}$ parameterize a map from the previous discrete state and continuous state to a distribution over the next discrete states using a softmax link function. The discrete state *z*_*t*_ determines the linear dynamical system used to generate the latent factors at any time *t*:

2 $${x}_{t}={A}_{{z}_{t}}{x}_{t-1}+{b}_{{z}_{t}}+{{\epsilon }}_{t}$$

where ${A}_{k}\in {{\mathbb{R}}}^{d\times d}$ is a dynamics matrix and ${b}_{k}\in {{\mathbb{R}}}^{D}$ is a bias vector, where *D* is the dimensionality of the latent space and ${{\epsilon }}_{t} \sim N(0,{Q}_{{z}_{t}})$ is a Gaussian-distributed noise (aka innovation) term.

Finally, we can recover the activity of recorded neurons by modelling activity as a linear noisy Gaussian observation ${y}_{t}\in {{\mathbb{R}}}^{N}$ where *N* is the number of recorded neurons:

3 $${y}_{t}=C{x}_{t}+d+{\delta }_{t}$$

For $C\in {{\mathbb{R}}}^{N\times D}$ and ${\delta }_{t} \sim N(0,S)$, a Gaussian noise term. Overall, the system parameters that rSLDS needs to learn consists of the state-transition dynamics, library of linear dynamical system matrices and neuron-specific emission parameters:

4 $$\theta =\{{\{{A}_{k}{b}_{k},{Q}_{k},\,{R}_{k},\,{r}_{k}\}}_{k=1}^{K},\,C,d,S\}$$

We evaluated model performance using both the evidence lower bound and the forwards simulation accuracy (Fig. 3a) described in Nair et al.^21^, as well as by calculating the variance explained by the model on data. In brief, given observed neural activity in the reduced neural state space at time *t*, we predicted the trajectory of population activity over an ensuing small time interval Δ*t* using the fit rSLDS model, then computed the mean squared error (MSE) between that trajectory and the observed data at time *t* + Δ*t*. This MSE was computed across all dimensions of the reduced latent space and repeated for all times *t* across cross-validation folds. This error metric is normalized to a 0–1 range in each animal across the whole recording to obtain a bounded measure of model performance. The forwards simulation accuracy can provide an intuition of where model performance drops during the recording. In addition to MSE, we also calculated the Pearson’s correlation coefficient (*R*^2^) between the predicted and observed data for each dimension following the forwards simulation. By taking the average correlation coefficient across dimensions, we obtained a quantitative estimate of variance explained by the rSLDS on observed data.

The number of states and dimensions used for the model are determined using fivefold cross-validation. Visualization of the dynamical system using principal component analysis is performed as previously described^21^.

For neural perturbation experiments, the rSLDS model was trained on data from unstimulated periods of time (that is, excluding data during and 20 s immediately after stimulation) and then tested on data from stimulated periods along with a 20-s post-stimulus period (Extended Data Fig. 5e,f).

The code used to fit the rSLDS on neural data is available in the SSM package: (https://github.com/lindermanlab/ssm).

### Estimation of time constants

We estimated the time constant of each dimension of linear dynamical systems using eigenvalues *λ*_*a*_ of the dynamics matrix of that system, as previously derived^39^:

5 $${\tau }_{a}=\left|\frac{1}{\log (\left|{\lambda }_{a}\right|)}\right|$$

### Decoding of behaviour using support vector machines

We trained frame-wise decoders to discriminate various pairs of behaviours as shown in Extended Data Fig. 4, from the population activity of all neurons during a mating interaction. We first created ‘trials’ from bouts of each behaviour by merging all bouts that were separated by less than 5 s and balanced data to ensure chance performance of the model to be 50%. We then trained a linear support vector machine to identify a decoding threshold that maximally separates the two behaviours and tested the accuracy of the trained decoder on held-out frames. ‘Shuffled’ decoder data were generated by setting the decoding threshold on the same trial, but with the behavioural annotations randomly assigned to each behavioural bout. We repeated shuffling 20 times. We report performances of actual and shuffled decoders as the average F1 score of the fit decoder, on data from all other trials for each mouse. For significance testing, the mean accuracy of the decoder trained on shuffled data was computed across mice, with shuffling repeated 1,000 times for each mouse.

### Dynamical system modelling using FORCE learning

We trained a recurrent neural network (RNN) to reproduce activity of individual neurons during mating interactions using FORCE as previously described^28,40^. The dynamics of each unit in the RNN is governed by the following equation:

$$\tau \frac{{\rm{d}}{x}_{i}}{{\rm{d}}t}=-{x}_{i}(t)+g(\mathop{\sum }\limits_{j=1}^{N}{J}_{ij}r({x}_{j}(t)))+H(t)$$

Here, *τ* is the time constant of the system (0.5 s as used previously^40^), *H* is the total external input to neurons (consisting of a weighted combination of male sniff, mounting and intromission), *J* is a heterogeneous matrix of recurrent connections whose strength is determined by the parameter *g*. For chaotic networks, we used *g* = 1.5 as used previously^28,40^. The elements of the matrix *J* are modified through recursive least squares as previously described^28,41^, by reducing an error term ${e}_{i}\left(t\right)={z}_{i}\left(t\right)-{f}_{i}(t)$. Here ${f}_{i}(t)$ is the experimental calcium trace and ${z}_{i}\left(t\right)={\sum }_{j}{J}_{{ij}}{r}_{j}(t)$. The network contains the same number of units as in the experimental data (between 100 and 200 neurons), and dynamics were solved using Euler’s method (d*t* = 0.05 s).

To estimate the fixed points of the RNN, we reverse engineered the trained RNN with fixed point analysis^42^ using gradient-based optimization. The estimated slow points of the dynamical system were then projected into the same neural state space as determined by the rSLDS to determine the similarity in attractor landscapes discovered by the two methods (Extended Data Fig. 5a,b).

### Modelling of integration dynamics to reveal inputs to the line attractor

To reveal the input received by the integration dimension (Extended Data Fig. 3r–u), we modelled activity of this dimension using a single-state linear dynamical system model as:

$${x}_{t}={A}_{z}{x}_{t-1}+{b}_{{z}_{t}}+{{\epsilon }}_{t}+W{u}_{t}$$

here *x* refers to weighted activity of the integration dimension and *W* is a matrix used to linearly combine behavioural inputs (male sniff, mounting and intromission) to the integration dimension. The model was fit as described above for the rSLDS, and model performance was evaluated using variance explained with cross-validation.

### Mechanistic modelling of spiking neural networks

We constructed a model population of *n* = 1,000 standard current-based leaky integrate-and-fire neurons as previously performed^43^. We modelled an excitatory spiking network with feedback inhibition designed to account for finite size effects and runaway excitation. In this network, each neuron has membrane potential *x*_*i*_ characterized by dynamics:

6 $${\tau }_{m}\frac{{\rm{d}}{x}_{i}}{{\rm{d}}t}=-{x}_{i}\left(t\right)+g\left(\mathop{\sum }\limits_{j=1}^{N}W{p}_{i}\left(t\right)-{g}_{{\rm{inh}}}{I}_{{\rm{inh}}}\left(t\right)\right)+{w}_{i}s\left(t\right)$$

where *τ*_*m*_ = 20 ms is the membrane time constant, *W* is the synaptic weight matrix, *s* is an input term representing external inputs and $p$ represents recurrent inputs. To model spiking, we set a threshold (*θ* = 0.1), such that when the membrane potential ${x}_{i}\left(t\right) > \theta$, ${x}_{i}\left(t\right)$ is set to zero and the instantaneous spiking rate ${r}_{i}\left(t\right)$ is set to 1.

Inhibition was modelled as recurrent inhibition from a single-graded input *I*_inh_ representing an inhibitory population that receives equal input from and provides equal input to all excitatory units. The dynamics of *I*_inh_ evolves as:

7 $${\tau }_{I}\frac{{\rm{d}}{I}_{{\rm{inh}}}}{{\rm{d}}t}={-I}_{{\rm{inh}}}\left(t\right)+\frac{1}{N}\mathop{\sum }\limits_{n=1}^{N}{r}_{N}(t),$$

where *τ*_*I*_ = 50 ms is the decay time constant for inhibitory currents.

We designed the synaptic connectivity matrix to include a subnetwork of 200 neurons (20% of the network), designated the integration subnetwork as suggested by empirical measurements, with a connectivity density of 12% as opposed to 1% in the remaining network. Weights of the overall network were sampled from a uniform distribution ${W}_{{ij}} \sim U(\mathrm{0,1}/\sqrt{N})$, whereas weights of the subnetwork were sampled as ${W}_{{ij}} \sim U(\mathrm{0,1}/\sqrt{{N}_{p}})$, where *Np* = 200.

External input was provided to the network as a step function consisting of 20 pulses at 10 interstimulus intervals (ISI). This stimulus drove a random 25% of neurons in each subnetwork.

Spiking-evoked input was modelled as a synaptic current with dynamics:

8 $${\tau }_{s}\frac{{\rm{d}}{p}_{i}}{{\rm{d}}t}=-{p}_{i}\left(t\right)+{r}_{i}\left(t\right),$$

where *τ*_*s*_ is the synaptic conductance time constant, set to 20 s for neurons in the integration subnetwork and 100 ms for remaining neurons in the network.

Model dynamics were simulated in discrete time using Euler’s method with a time step of 1 ms and a small Gaussian noise term ${\eta }_{i} \sim N(\mathrm{0,1})/5$ were added at each time step. We used *g* = 2.5 and varied *g*_inh_ = 4.25 as suggested by measurements of inhibitory input to the VMHvl^44^ and used previously^43^. To simulate hypothesis 1 in Extended Data Fig. 8, we set the synaptic time constant for integration neurons to 100 ms. To simulate hypothesis 2, we changed the gain associated with input to each subnetwork, decreasing this quantity for the integration subnetwork by 50% and increasing the same for the remaining neurons by 50%.

### Calculation of ACHW

We computed ACHWs by calculating the autocorrelation function for each neuronal time series data (*y*_*t*_) for a set of lags as previously described^22^. In brief, for a time series (*y*_*t*_), the autocorrelation for lag *k* is:

$${r}_{k}=\frac{{c}_{k}}{{c}_{0}}$$

where *c*_*k*_ is defined as:

$${c}_{k}=\frac{1}{T}\mathop{\sum }\limits_{t=1}^{T-k}(\,{y}_{t}-\bar{y})(\,{y}_{t+k}-\bar{y})$$

and *c*_0_ is the sample variance of the data. The half-width is found for each neuron as the point where the autocorrelation function reaches a value of 0.5.

### Partial least-squares regression to identify integration dynamics

To identify the integration dimension using an independent method, we also used partial least-squares regression. Towards this, all traces were concatenated and regressed against a 1 × **T** vector designed such that the vector shows ramping activity upon entry of the male intruder (Extended Data Fig. 3d,e).

### Statistics and reproducibility

All experiments were conducted using 2–4 cohorts of animals. The results were reproducible across cohorts and combined for the final analysis.

### Reporting summary

Further information on research design is available in the Nature Portfolio Reporting Summary linked to this article.

## Online content

Any methods, additional references, Nature Portfolio reporting summaries, source data, extended data, supplementary information, acknowledgements, peer review information; details of author contributions and competing interests; and statements of data and code availability are available at 10.1038/s41586-024-07916-w.

## Supplementary information

Reporting Summary

Supplemental Video 1 Mating assay with intromission behaviour and high sexual receptivity in female mice. Mating assay where the female mouse exhibits high sexual receptivity which is accompanied by intromission behaviour by the male mouse.

Supplementary Video 2 Mating assay with intromission behaviour and low sexual receptivity in female mice. Mating assay where the female mouse exhibits low sexual receptivity which is accompanied by intromission behaviour by the male mouse.

## Data Availability

Data pertaining to this Article have been deposited in the DANDI repository with the accession code 001097.

## Code availability

The code to analyse the rSLDS models is available at GitHub (https://github.com/lindermanlab/ssm). The rSLDS model weights and parameters have also been deposited in the DANDI with the accession code 001097.

## References

[1] Jennings, K. J. & de Lecea, L. Neural and hormonal control of sexual behavior. *Endocrinology* **161**, bqaa150 (2020). 32845294 10.1210/endocr/bqaa150 PMC7507403

[2] Gutierrez-Castellanos, N., Husain, B. F. A., Dias, I. C. & Lima, S. Q. Neural and behavioral plasticity across the female reproductive cycle. *Trends Endocrinol. Metab.*10.1016/j.tem.2022.09.001 (2022). 10.1016/j.tem.2022.09.001 36253276

[3] Yin, L. & Lin, D. Neural control of female sexual behaviors. *Horm. Behav.* **151**, 105339 (2023). 36878049 10.1016/j.yhbeh.2023.105339 PMC10133197

[4] Lenschow, C. & Lima, S. Q. In the mood for sex: neural circuits for reproduction. *Curr. Opin. Neurobiol.* **60**, 155–168 (2020). 31901622 10.1016/j.conb.2019.12.001

[5] Liu, M., Kim, D.-W., Zeng, H. & Anderson, D. J. Make war not love: the neural substrate underlying a state-dependent switch in female social behavior. *Neuron* **110**, 841–856.e6 (2022). 34982958 10.1016/j.neuron.2021.12.002 PMC8897222

[6] Yin, L. et al. VMHvll^Cckar^ cells dynamically control female sexual behaviors over the reproductive cycle. *Neuron* **110**, 3000–3017.e8 (2022). 35896109 10.1016/j.neuron.2022.06.026 PMC9509472

[7] Pfaff, D. W., Diakow, C., Zigmond, R. E. & Kow, L. M. Neural and hormonal determinants of female mating behavior in rats. *Neuroscience* **3**, 621–646 (1974).

[8] Pfaff, D. W., Gagnidze, K. & Hunter, R. G. Molecular endocrinology of female reproductive behavior. *Mol. Cell. Endocrinol.* **467**, 14–20 (2018). 29100890 10.1016/j.mce.2017.10.019

[9] Rodriguez-Sierra, J. F., Crowley, W. R. & Komisaruk, B. R. Vaginal stimulation in rats induces prolonged lordosis responsiveness and sexual receptivity. *J. Comp. Physiol. Psychol.* **89**, 79–85 (1975). 1159114 10.1037/h0076442

[10] Boyd, K. L., Muehlenbachs, A., Rendi, M. H., Garcia, R. L. & Gibson-Corley, K. N. *Female Reproductive System* (Academic Press, 2018).

[11] Micevych, P. E. & Meisel, R. L. Integrating neural circuits controlling female sexual behavior. *Front. Syst. Neurosci.* **11**, 42 (2017). 28642689 10.3389/fnsys.2017.00042 PMC5462959

[12] Pfaff, D. W. & Sakuma, Y. Deficit in the lordosis reflex of female rats caused by lesions in the ventromedial nucleus of the hypothalamus. *J. Physiol.* **288**, 203–210 (1979). 469716 PMC1281422

[13] Pfaff, D. W. & Sakuma, Y. Facilitation of the lordosis reflex of female rats from the ventromedial nucleus of the hypothalamus. *J. Physiol.* **288**, 189–202 (1979). 469715 PMC1281421

[14] Yang, C. F. et al. Sexually dimorphic neurons in the ventromedial hypothalamus govern mating in both sexes and aggression in males. *Cell* **153**, 896–909 (2013). 23663785 10.1016/j.cell.2013.04.017 PMC3767768

[15] Hashikawa, K. et al. Esr1^+^ cells in the ventromedial hypothalamus control female aggression. *Nat. Neurosci.* **20**, 1580–1590 (2017). 28920934 10.1038/nn.4644 PMC5953764

[16] Inoue, S. et al. Periodic remodeling in a neural circuit governs timing of female sexual behavior. *Cell* **179**, 1393–1408.e16 (2019). 31735496 10.1016/j.cell.2019.10.025 PMC7096331

[17] Knoedler, J. R. et al. A functional cellular framework for sex and estrous cycle-dependent gene expression and behavior. *Cell* **185**, 654–671.e22 (2022). 35065713 10.1016/j.cell.2021.12.031 PMC8956134

[18] Ziv, Y. et al. Long-term dynamics of CA1 hippocampal place codes. *Nat. Neurosci.* **16**, 264–266 (2013). 23396101 10.1038/nn.3329 PMC3784308

[19] Linderman, S. W. et al. Bayesian learning and inference in recurrent switching linear dynamical systems. In *Proc. 20th Int. Conf. Artif. Intell. Stat. AISTATS 2017* **54**, 914–922 (PMLR, 2017).

[20] Beach, F. A. Sexual attractivity, proceptivity, and receptivity in female mammals. *Horm. Behav.* **7**, 105–138 (1976). 819345 10.1016/0018-506x(76)90008-8

[21] Nair, A. et al. An approximate line attractor in the hypothalamus that encodes an aggressive internal state. *Cell* **186**, 178–193 (2022). 10.1016/j.cell.2022.11.027 PMC9990527 36608653

[22] Remedios, R. et al. Social behaviour shapes hypothalamic neural ensemble representations of conspecific sex. *Nature* **550**, 388–392 (2017). 29052632 10.1038/nature23885 PMC5674977

[23] Pillow, J. W. et al. Spatio-temporal correlations and visual signalling in a complete neuronal population. *Nature* **454**, 995–999 (2008). 18650810 10.1038/nature07140 PMC2684455

[24] Weissbourd, B. et al. A genetically tractable jellyfish model for systems and evolutionary neuroscience. *Cell* **184**, 5854–5868.e20 (2021). 34822783 10.1016/j.cell.2021.10.021 PMC8629132

[25] Cavanagh, S. E., Towers, J. P., Wallis, J. D., Hunt, L. T. & Kennerley, S. W. Reconciling persistent and dynamic hypotheses of working memory coding in prefrontal cortex. *Nat. Commun.* **9**, 3498 (2018). 30158519 10.1038/s41467-018-05873-3 PMC6115433

[26] Murray, J. D. et al. A hierarchy of intrinsic timescales across primate cortex. *Nat. Neurosci.* **17**, 1661–1663 (2014). 25383900 10.1038/nn.3862 PMC4241138

[27] Mante, V., Sussillo, D., Shenoy, K. V. & Newsome, W. T. Context-dependent computation by recurrent dynamics in prefrontal cortex. *Nature* **503**, 78–84 (2013). 24201281 10.1038/nature12742 PMC4121670

[28] Rajan, K., Harvey, C. D. D. & Tank, D. W. W. Recurrent network models of sequence generation and memory. *Neuron* **90**, 128–142 (2016). 26971945 10.1016/j.neuron.2016.02.009 PMC4824643

[29] Khona, M. & Fiete, I. R. Attractor and integrator networks in the brain. *Nat. Rev. Neurosci.* **23**, 744–766 (2022). 36329249 10.1038/s41583-022-00642-0

[30] Kunwar, P. S. et al. Ventromedial hypothalamic neurons control a defensive emotion state. *eLife* **4**, e06633 (2015). 10.7554/eLife.06633 PMC4379496 25748136

[31] Wang, L., Chen, I. Z. & Lin, D. Collateral pathways from the ventromedial hypothalamus mediate defensive behaviors. *Neuron* **85**, 1344–1358 (2015). 25754823 10.1016/j.neuron.2014.12.025 PMC4368499

[32] Kim, D.-W. et al. Multimodal analysis of cell types in a hypothalamic node controlling social behavior. *Cell* **179**, 713–728.e17 (2019). 31626771 10.1016/j.cell.2019.09.020 PMC7534821

[33] Lo, L. et al. Connectional architecture of a mouse hypothalamic circuit node controlling social behavior. *Proc. Natl Acad. Sci. USA* **116**, 7503–7512 (2019). 30898882 10.1073/pnas.1817503116 PMC6462064

[34] Knoedler, J. R. & Shah, N. M. Molecular mechanisms underlying sexual differentiation of the nervous system. *Curr. Opin. Neurobiol.* **53**, 192–197 (2018). 30316066 10.1016/j.conb.2018.09.005 PMC6347421

[35] Karigo, T. et al. Distinct hypothalamic control of same- and opposite-sex mounting behaviour in mice. *Nature* **589**, 258–263 (2021). 33268894 10.1038/s41586-020-2995-0 PMC7899581

[36] Yang, B., Karigo, T. & Anderson, D. J. Transformations of neural representations in a social behaviour network. *Nature* **608**, 741–749 (2022). 35922505 10.1038/s41586-022-05057-6 PMC9529293

[37] Hong, W. et al. Automated measurement of mouse social behaviors using depth sensing, video tracking, and machine learning. *Proc. Natl Acad. Sci. USA* **112**, E5351–E5360 (2015). 26354123 10.1073/pnas.1515982112 PMC4586844

[38] Linderman, S. W. et al. Bayesian learning and inference in recurrent switching linear dynamical systems. In *Proc. 20th International Conference on Artificial Intelligence and Statistics* 54, 914–922 (PMLR, 2017).

[39] Maheswaranathan, N., Williams, A., Golub, M., Ganguli, S. & Sussillo, D. Reverse engineering recurrent networks for sentiment classification reveals line attractor dynamics. *Adv. Neural Inf. Process. Syst.* **32**, 15696–15705 (2019). PMC7416638 32782423

[40] Hadjiabadi, D. et al. Maximally selective single-cell target for circuit control in epilepsy models. *Neuron* **109**, 2556–2572.e6 (2021). 34197732 10.1016/j.neuron.2021.06.007 PMC8448204

[41] Sussillo, D. & Abbott, L. F. Generating coherent patterns of activity from chaotic neural networks. *Neuron* **63**, 544–557 (2009). 19709635 10.1016/j.neuron.2009.07.018 PMC2756108

[42] Sussillo, D. & Barak, O. Opening the black box: low-dimensional dynamics in high-dimensional recurrent neural networks. *Neural Comput.* **25**, 626–649 (2013). 23272922 10.1162/NECO_a_00409

[43] Kennedy, A. et al. Stimulus-specific hypothalamic encoding of a persistent defensive state. *Nature* **586**, 730–734 (2020). 32939094 10.1038/s41586-020-2728-4 PMC7606611

[44] Yamamoto, R., Ahmed, N., Ito, T., Gungor, N. Z. & Pare, D. Optogenetic study of anterior BNST and basomedial amygdala projections to the ventromedial hypothalamus. *eNeuro* **5**, ENEURO.0204-18.2018 (2018). 29971248 10.1523/ENEURO.0204-18.2018 PMC6027956

