# Supplementary material for: Encoding of female mating dynamics by a hypothalamic line attractor
Source: Nature. 2024 Aug 14;634(8035):901–9. doi: 10.1038/s41586-024-07916-w (PMC11499253; doi:10.1038/s41586-024-07916-w)
Supplement: Supplementary file 1 — Reporting Summary [file 41586_2024_7916_MOESM1_ESM.pdf]

nature portfolio

Corresponding author(s): David J. Anderson

Last updated by author(s): Jul 29, 2024

Reporting Summary

Nature Portfolio wishes to improve the reproducibility of the work that we publish. This form provides structure for consistency and transparency in reporting. For further information on Nature Portfolio policies, see our Editorial Policies and the Editorial Policy Checklist.

Statistics

For all statistical analyses, confirm that the following items are present in the figure legend, table legend, main text, or Methods section.

n/a	Confirmed	
☐	☒	The exact sample size (n) for each experimental group/condition, given as a discrete number and unit of measurement
☐	☒	A statement on whether measurements were taken from distinct samples or whether the same sample was measured repeatedly
☐	☒	The statistical test(s) used AND whether they are one- or two-sided<br>*Only common tests should be described solely by name; describe more complex techniques in the Methods section.*
☐	☒	A description of all covariates tested
☐	☒	A description of any assumptions or corrections, such as tests of normality and adjustment for multiple comparisons
☐	☒	A full description of the statistical parameters including central tendency (e.g. means) or other basic estimates (e.g. regression coefficient) AND variation (e.g. standard deviation) or associated estimates of uncertainty (e.g. confidence intervals)
☐	☒	For null hypothesis testing, the test statistic (e.g. F, t, r) with confidence intervals, effect sizes, degrees of freedom and P value noted<br>*Give P values as exact values whenever suitable.*
☐	☒	For Bayesian analysis, information on the choice of priors and Markov chain Monte Carlo settings
☐	☒	For hierarchical and complex designs, identification of the appropriate level for tests and full reporting of outcomes
☐	☒	Estimates of effect sizes (e.g. Cohen's d, Pearson's r), indicating how they were calculated

Our web collection on statistics for biologists contains articles on many of the points above.

Software and code

Policy information about availability of computer code

Data collection	We listed all softwares used in the experiments and for analysis in the Methods section. StreamPix7 (Norpix) was used to synchronize video and neural recordings. A custom MATLAB-based behavior annotation interface was used to manually annotate behavior videos. Synapse software (Tucker Davis Technologies) was used to collect the fiberphotometry signal. FluoView (v Olympus) was used to to capture Confocal images. Inscopix Data Acquisition Software (IDAS v2.0) was used to collect micro-endoscope data.
Data analysis	Inscopix Data Processing Software (IDPS v1.7) was used to for preprocessing and motion correction. Customized Python (v3.8) code was used for analyzing behavior data and fiberphotometry data. GraphPad (v8) was used to perform statistics for behavior data.<br><br>Code to analysis code for rSLDS models is available here: https://github.com/lindermanlab/ssm. rSLDS model weights and parameters have also been deposited in the DANDI repo with the accession number: DANDI:001097

For manuscripts utilizing custom algorithms or software that are central to the research but not yet described in published literature, software must be made available to editors and reviewers. We strongly encourage code deposition in a community repository (e.g. GitHub). See the Nature Portfolio guidelines for submitting code & software for further information.

Data

Policy information about availability of data

All manuscripts must include a data availability statement. This statement should provide the following information, where applicable:

- Accession codes, unique identifiers, or web links for publicly available datasets
- A description of any restrictions on data availability
- For clinical datasets or third party data, please ensure that the statement adheres to our policy

Data pertaining to this manuscript has been deposited in DANDI, with the following accession number: DANDI:001097

Research involving human participants, their data, or biological material

Policy information about studies with human participants or human data. See also policy information about sex, gender (identity/presentation), and sexual orientation and race, ethnicity and racism.

Reporting on sex and gender	Not applicable.
Reporting on race, ethnicity, or other socially relevant groupings	Not applicable.
Population characteristics	Not applicable.
Recruitment	Not applicable.
Ethics oversight	Not applicable.

Note that full information on the approval of the study protocol must also be provided in the manuscript.

Field-specific reporting

Please select the one below that is the best fit for your research. If you are not sure, read the appropriate sections before making your selection.

☒ Life sciences ☐ Behavioural & social sciences ☐ Ecological, evolutionary & environmental sciences

For a reference copy of the document with all sections, see nature.com/documents/nr-reporting-summary-flat.pdf

Life sciences study design

All studies must disclose on these points even when the disclosure is negative.

Sample size	We have determined that our size is sufficient to achieve the objectives of our study while maintaining statistical power and ethical standards.
Data exclusions	We did not exclude any data.
Replication	All experiments were conducted using 2 to 4 cohorts of animals. The results were reproducible across cohorts and combined for the final analysis.
Randomization	This is not applicable for this study because of no data grouping.
Blinding	This is not applicable for this study because of no data grouping.

Reporting for specific materials, systems and methods

We require information from authors about some types of materials, experimental systems and methods used in many studies. Here, indicate whether each material, system or method listed is relevant to your study. If you are not sure if a list item applies to your research, read the appropriate section before selecting a response.

Materials & experimental systems

n/a	Involved in the study
☒	☐ Antibodies
☒	☐ Eukaryotic cell lines
☒	☐ Palaeontology and archaeology
☐	☒ Animals and other organisms
☒	☐ Clinical data
☒	☐ Dual use research of concern
☒	☐ Plants

Methods

n/a	Involved in the study
☒	☐ ChIP-seq
☒	☐ Flow cytometry
☒	☐ MRI-based neuroimaging

Animals and other research organisms

Policy information about studies involving animals; ARRIVE guidelines recommended for reporting animal research, and Sex and Gender in Research

Laboratory animals	All mice used in this research aged 2-5 months. Wild type C57BL/6N mice were bred at Caltech or purchased from Charles River Laboratory. Npy2r-cre mice (Jackson Laboratory stock no. 029285) (=N1), Esr1-cre mice (Jackson Laboratory stock no. 017913), Esr1-flpo mice (Jackson Laboratory stock no. 036028) (>N10), Sf1-cre mice (Jackson Laboratory stock no. 012462) were back-crossed into the C57BL/6N background and bred at Caltech.
Wild animals	The study did not involve wild animals.
Reporting on sex	Females were used as subjects.
Field-collected samples	The study did not involve samples collected from the field.
Ethics oversight	All experimental procedures involving the use of live mice or their tissues were carried out in accordance with NIH guidelines and approved by the Institute Animal Care and Use Committee (IACUC) and the Institute Biosafety Committee (IBC) at the California Institute of Technology (Caltech).

Note that full information on the approval of the study protocol must also be provided in the manuscript.

Plants

Seed stocks	Not applicable.
Novel plant genotypes	Not applicable.
Authentication	Not applicable.
